# Allylation of Orthoquinones Towards Annulated Polycyclic Aromatic Systems

**DOI:** 10.3390/molecules23082043

**Published:** 2018-08-15

**Authors:** Mariusz Kędziorek, Liliana Dobrzańska

**Affiliations:** 1 Faculty of Chemistry, University of Warsaw, Żwirki i Wigury 101, 02-089 Warsaw, Poland; 2 Faculty of Chemistry, Nicolaus Copernicus University in Toruń, Gagarina 7, 87-100 Toruń, Poland; lianger@umk.pl

**Keywords:** allylation, polycyclic aromatic compounds (PACs), orthoquinones, pinacol rearrangement, Wagner-Meerwein rearrangement, metathesis, tris(pentafluorophenyl)borane

## Abstract

Promising results of an efficient and convenient strategy for the annulation of polycyclic aromatic compounds (PACs), employing orthoquinones as starting material and comprising allylation, pinacol rearrangement, ring-closing metathesis (RCM), and one-pot reduction followed by Wagner-Meerwein rearrangement, are presented. The strategy involves introducing triallylborane prepared in situ in the allylation step. Moreover, a novel expedient method for the preparation of 9,10-diallylphenanthrene was introduced.

## 1. Introduction

Polycyclic aromatic compounds (PACs) have attracted particular interest because of their unique chemical and physical properties and consequently promising applications in organic electronics, such as light-emitting diodes (LEDs), field-effect transistors (FETs), and photovoltaic cells [1,2,3,4]. In view of these, the development and elaboration of effective strategies for the preparation of PACs remain of significance [5].

The functionalization of polycyclic aromatic hydrocarbons is a difficult task. Problems with insufficient differentiation of chemical reactivity of several aromatic C-H bonds usually lead to a complex mixture of regioisomers. On the other hand, known regioselective reactions are restricted to certain positions in the aromatic core, which is one of the reasons that the number of described synthetic procedures is limited. The most popular synthetic strategies start from simple aromatic precursors (also with substituents) and exploit oxidative aromatic coupling as the key step leading to fused aromatic systems [6,7,8,9,10,11]. However, this approach is usually limited to reasonably electron-rich aromatic systems. Thus, among known compounds, those with alkyl or alkoxyl substituents are predominant.

Herein, we present preliminary results towards the development of an efficient method for the synthesis of polycyclic aromatic systems starting from polycyclic orthoquinones. The presented synthetic strategy relies on the following four-step sequence: (i) addition of allyl substituents to carbonyl groups to produce a 1,2-diol; (ii) subsequent conversion to α,α-diallyl ketone by pinacol rearrangement; (iii) ring-closing metathesis of allyl groups with the formation of spirocyclopentene; and finally (iv) a one-pot procedure comprising reduction of the keto group and Wagner-Meerwein rearrangement, providing a cyclohexene-annuleted polycyclic aromatic compound. This approach also facilitates the synthesis of fully aromatized analogues with the use of an oxidant [12]. The cyclohexene ring can also be utilized in Diels-Alder cycloaddition [13,14].

## 2. Results and Discussion

### 2.1. Synthesis of 1,2-Diols via Addition of Allyl Substituents to Carbonyl Groups in Orthoquinones

**Scheme 1:**
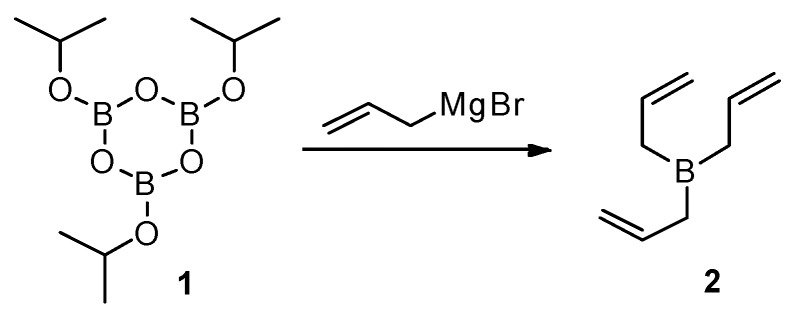
In situ generation of triallylborane (**2**).

Addition of allyl substituents to carbonyl groups in orthoquinones can be performed through a Grignard reaction. However, the yields are not satisfactory, and undesired products are formed [15,16]. Much better results are accomplished with triallylborane (**2**) [17]. Considerable advantages of this reagent are diminished by the fact that triallylborane (**2**) is an air sensitive and pyrophoric liquid, thus its use suffers from the difficulties involved with its preparation (isolation) and subsequent storage and handling [18,19,20]. Therefore, in situ generation of compound **2** would be much more convenient, for which we introduced a methodology based on reaction of 2,4,6-triisopropoxy-1,3,5,2,4,6-trioxatriborinane (**1**) with allylmagnesium bromide (Scheme 1).

Boron compound **1** can be easily obtained in pure form [21]. Because this is a crystalline compound, purification via crystallization is very convenient, and also its storage and handling are not problematic. Compound **1** smoothly reacts with an allylmagnesium bromide solution to give triallylborane (**2**) [22]. Prepared in this way, triallylborane (**2**) allows to obtain products of formal addition of allyl anion to keto groups of orthoquinones with very high yields (Table 1).

### 2.2. Pinacol Rearrangement of 1,2-Diol to α,α-Diallyl Ketone

**Scheme 2:**
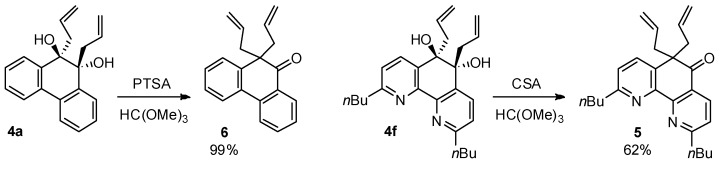
Pinacol rearrangement of diols.

Pinacol rearrangement of diol **4a** was achieved in acidic conditions with *p*-toluenesulfonic acid (PTSA) in the presence of trimethyl orthoformate yielding 10,10-diallylphenanthren-9(10*H*)-one (**6**, 99%), while diol **4f** was transformed in the presence of camphorosulfonic acid (CSA) (Scheme 2).

**Figure 1:**
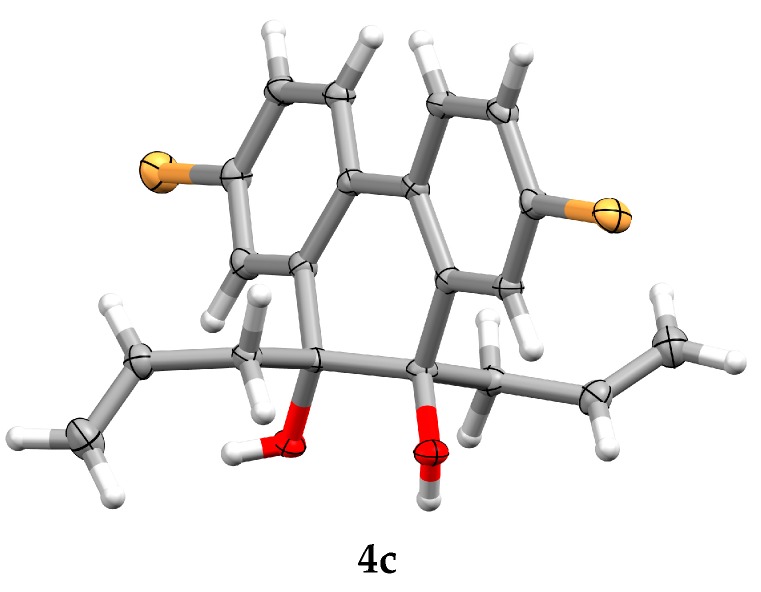
Molecular structure of **4c**. Displacement ellipsoids are drawn at the 50% probability level.

Pinacol rearrangement can be mediated by trimethyl orthoformate via a cyclic ortho ester intermediate [23]. However, it was shown by X-ray analysis that the 1,2-diols obtained in the reaction of orthoquinones with triallylborane (**2**) have the trans configuration (Figure 1). From a mechanistic point of view, protonation of the hydroxyl group with subsequent release of a water molecule is reversible [24]. Thus, during the course of the reaction, the formation of 1,2-diol molecules with the cis configuration is possible, but direct formation of a cyclic ortho ester intermediate is unlikely and trimethyl orthoformate presumably acts primarily as a dehydrating agent, shifting the reaction towards the product.

### 2.3. Ring Closing Metathesis (RCM)

**Scheme 3:**
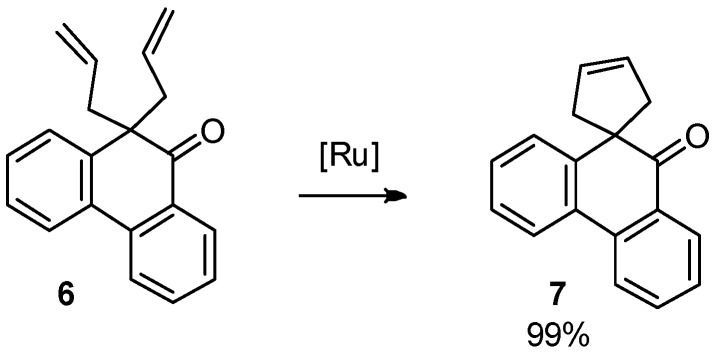
Ring closing metathesis in the presence of ruthenium metathesis catalyst.

As observed experimentally, 1,2-diols obtained in the reaction of orthoquinones **3** with triallylborane (**2**) don′t undergo RCM reaction to form a cyclohexene ring (from the reaction mixture, only unreacted 1,2-diol was isolated). This can be easily explained by the relative orientation of the allyl groups (trans-axial, Figure 1). However, the ketone obtained in the pinacol rearrangement easily reacts in the presence of commercial Umicore™ M2 ruthenium metathesis catalyst with the exclusive formation of a spirocyclopentene ring (Scheme 3).

### 2.4. One-pot Reduction of Keto Group and Wagner-Meerwein Rearrangement

**Scheme 4:**
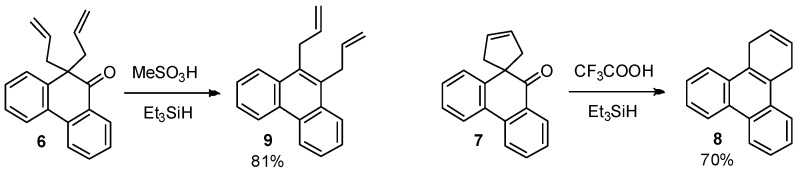
One-pot reduction and Wagner-Meerwein rearrangement.

The product of the pinacol rearrangement with allyl groups **6** was transformed via a straightforward one-pot procedure to 9,10-diallylphenanthrene (**9**), while ketone **7** (with cyclopentene ring) yielded the appropriate polycyclic aromatic compound **8** (Scheme 4).

This was accomplished by reduction with Et_3_SiH in acidic conditions [25]. The reaction performed in trifluoroacetic acid has the advantage that the product precipitates from the reaction mixture and can easily be isolated in pure form.

### 2.5. Direct Dehydroxylation of 9,10-Diallyl-9,10-dihydrophenanthrene-9,10-diol (***4a***) to 9,10-Diallylphenanthrene (***9***)

**Scheme 5:**
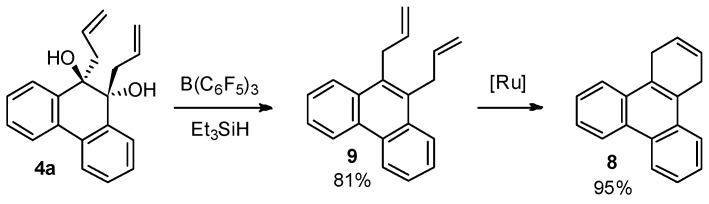
Direct dehydroxylation of 9,10-diallyl-9,10-dihydrophenanthrene-9,10-diol (**4a**) and subsequent ring closing metathesis.

Recent studies have demonstrated that reduction of vicinal aliphatic diols mediated by the catalyst tris(pentafluorophenyl)borane (B(C_6_F_5_)_3_) in the presence of silanes afforded rearranged alcohols [26]. This catalytic system is also active in the reduction of primary, secondary, and tertiary alcohols to the corresponding hydrocarbons [27]. During the performed studies we discovered that in the case of 9,10-diallyl-9,10-dihydrophenanthrene-9,10-diol (**4a**), a catalytical amount of B(C_6_F_5_)_3_ in the presence of triethylsilane causes direct dehydroxylation, yielding 9,10-diallylphenanthrene (**9**) which easily reacts to the polycliclic aromatic compound **8** in the presence of commercial Umicore™ M2 ruthenium metathesis catalyst (Scheme 5).

**Scheme 6:**
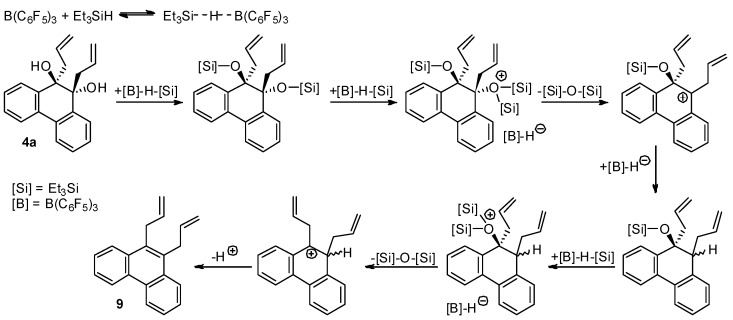
Plausible mechanism of direct dehydroxylation of 9,10-diallyl-9,10-dihydrophenanthrene-9,10-diol (**4a**).

The question arises by which mechanism this novel transformation proceeds. To check whether pinacol rearrangement is the first step in this transformation, the reaction of 10,10-diallylphenanthren-9(10*H*)-one (**6**) with triethylsilane in the presence of a catalytic amount of B(C_6_F_5_)_3_ was performed. However, the product isolated from this reaction was not 9,10-diallylphenanthrene (**9**). This experiment unambiguously excludes that this transformation proceeds via pinacol rearrangement. This was further confirmed by the unreactivity of 2,2,4,4,6,6-hexaallylcyclohexane-1,3,5-trione [28] for which the formation of hexaallylbenzene was expected under these conditions. We assume that the reaction begins with the bissilylation of diol **4a** with subsequent formation of a disilyl oxonium ion. This is followed by cleavage of the C‒O bond to form a tertiary benzylic carbocation, that is then reduced by hydride transfer. Further silylation forms a second disilyl oxonium ion and subsequent cleavage of the C‒O bond results in another tertiary benzylic carbocation. Finally, a double bond is formed through release of a proton (Scheme 6).

The proposed reaction mechanism implies that four equivalents of silane are necessary to fully transform diol **4a** to compound **9**. However, we have experimentally checked that this stoichiometry leads to a lower yield of product **9** than with 3.2 equivalents of silane as the formation of side products is more significant.

We were interested in examining other substrates for this transformation. The performed studies revealed that 2,7-dibromo-9,10-diallyl-9,10-dihydrophenanthrene-9,10-diol (**4c**) doesn′t react and that a complex mixture was obtained from the reaction with 3,6-dibromo-9,10-diallyl-9,10-dihydrophenanthrene-9,10-diol (**4b**) or 4,5-diallyl-4,5-dihydropyrene-4,5-diol (**4d**). Also, with an equimolar mixture of 9,10-diallyl-9,10-dihydrophenanthrene-9,10-diol (**4a**) and 2,7-dibromo-9,10-diallyl-9,10-dihydrophenanthrene-9,10-diol (**4c**), there is no reaction, suggesting that the catalyst B(C_6_F_5_)_3_ is inhibited by bromoderivative **4c**.

## 3. Materials and Methods

### 3.1. General Methods

Flash chromatography was performed using silica gel 60 (230–400 mesh). Analytical thin-layer chromatography (TLC) was performed using silica gel 60 F254 precoated plates (Merck, Darmstadt, Germany) (0.25 mm thickness) with a fluorescent indicator. Visualization of TLC plates was performed by UV light (Carl Roth, Karsruhe, Germany). NMR spectra were recorded on an Agilent 400-MR DD2 400 MHz spectrometer (Agilent Technologies, Santa Clara, CA, USA). NMR chemical shifts are reported in ppm and referred to residual solvent peaks at respectively 7.26 and 77.16 ppm for ^1^H and ^13^C in CDCl_3_, and 2.50 and 39.52 ppm for ^1^H and ^13^C in DMSO-*d_6_*. The following abbreviations are used in reporting NMR data: s (singlet), d (doublet), t (triplet), q (quartet), sep (septet), m (multiplet), br (broad). Unless otherwise stated, all coupling constants (*J*) are between protons through three bonds and expressed in Hz. Spectra are reported as follows: chemical shift (*δ*, ppm), multiplicity, integration, coupling constants (Hz). Melting points were recorded on an OptiMelt SRS apparatus. Electrospray mass spectra (ESI) were recorded on a Waters AutoSpec Premier mass spectrometer (Waters, Milford, MA, USA). Elemental analyses were performed on a Perkin Elmer CHN 2400 instrument.

2,4,6-Triisopropoxy-1,3,5,2,4,6-trioxatriborinane (**1**) was prepared according to literature procedure [21]. ^1^H-NMR (400 MHz, CDCl_3_): *δ* 4.55 (sep, 3H, 6.2 Hz), 1.21 (d, 18H, 6.2 Hz); ^13^C-NMR (100 MHz, CDCl_3_): *δ* 67.0, 24.3; ^11^B-NMR (128 MHz, CDCl_3_): *δ* 18.7.

2,9-Dibutyl-1,10-phenanthroline-5,6-dione (**3f**) was prepared according to literature procedure [29]. ^1^H-NMR (400 MHz, CDCl_3_): *δ* 8.36 (d, 2H, 8.4 Hz), 7.40 (d, 2H, 8.4 Hz), 3.10–3.06 (m, 4H), 1.88–1.81 (m, 4H), 1.53–1.43 (m, 4H), 0.99 (t, 6H, 7.2 Hz); ^13^C-NMR (100 MHz, CDCl_3_): *δ* 179.2, 171.1, 152.9, 137.4, 126.3, 124.5, 39.2, 31.3, 22.8, 14.1.

Compounds: 3,6-dibromo-phenanthrene-9,10-dione (**3b**) [30], 2,7-dibromo-phenanthrene-9,10-dione (**3c**) [31], pyrene-4,5-dione (**3d**) [32], and 9,10-dibromopyrene-4,5-dione (**3e**) [33] were synthesized as previously reported and their characterization data agreed with those reported. The Grignard reagent was prepared from the allyl bromide and their solution titrated immediately prior to use according to standard procedure [34].

Copies of the NMR spectra of compounds **1**, **3f**, **4a–f** and **5**–**9** can be found in the Supplementary Materials.

### 3.2. General Procedure for In Situ Triallylborane Generation and Their Reaction with the Quinone Carbonyl Groups

To the stirred solution of 2,4,6-triisopropoxy-1,3,5,2,4,6-trioxatriborinane (**1**) (383 mg, 1.49 mmol) in anhydrous Et_2_O (20 mL) under argon at 0 °C, a solution of allylmagnesium bromide (1.2 M in Et_2_O, 10 mL, 12 mmol) was added over 5 min. Formation of white precipitate was observed. Next, the mixture was warmed up to 22 °C. After 15 min this mixture was cooled down to 0 °C and quinone **3** (3.00 mmol) was added. Next, the reaction mixture was warmed up to 22 °C and after 30 min again cooled down to 0 °C and saturated solution of NH_4_Cl_aq_ (100 mL) was added. The product was extracted with ethyl acetate (3 × 25 mL) and isolated by column chromatography.

*9,10-Diallyl-9,10-dihydrophenanthrene-9,10-diol* (**4a**): yield 93%, colorless crystals, m.p. 72–74 °C (lit. [35] 73–75 °C); ^1^H-NMR (400 MHz, CDCl_3_): *δ* 7.71 (dd, 2H, 7.2, 1.6 Hz), 7.50 (dd, 2H, 7.6, 1.6 Hz), 7.38–7.30 (m, 4H), 5.62–5.51 (m, 2H), 5.07–4.95 (m, 4H), 2.65–2.59 (m, 2H), 2.51 (br s, 2xOH), 2.29–2.24 (m, 2H); ^13^C-NMR (100 MHz, CDCl_3_): δ 139.8, 134.0, 132.1, 127.9, 127.8, 125.5, 123.8, 119.4, 78.2, 40.1.

*9,10-Diallyl-3,6-dibromo-9,10-dihydrophenanthrene-9,10-diol* (**4b**): yield 85%, colorless crystals, m.p. 140–146 °C; ^1^H-NMR (400 MHz, CDCl_3_): *δ* 7.8 (d, 2H, 2.0 Hz), 7.47 (dd, 2H, 8.0, 2.0 Hz), 7.38 (d, 2H, 8.0 Hz), 5.56–5.46 (m, 2H), 5.09–4.94 (m, 4H), 2.62–2.56 (m, 2H), 2.51 (s, 2xOH), 2.22–2.17 (m, 2H); ^13^C-NMR (100 MHz, CDCl_3_): *δ* 139.1, 133.2, 132.9, 131.3, 127.7, 126.9, 122.2, 120.2, 77.8, 40.0; HRMS (ES+) calcd for C_20_H_18_Br_2_O_2_Na: 472.9552, found: 472.9568.

*9,10-Diallyl-2,7-dibromo-9,10-dihydrophenanthrene-9,10-diol* (**4c**): yield 94%, light yellow crystals, m.p. 145–147 °C; ^1^H-NMR (400 MHz, CDCl_3_): *δ* 7.65 (d, 2H, 2.0 Hz), 7.53–7.46 (m, 4H), 5.60–5.49 (m, 2H), 5.13–4.97 (m, 4H), 2.61 (dd, 2H, 14.0, 9.2 Hz), 2.24 (s, 2xOH), 2.24–2.18 (m, 2H); ^13^C-NMR (100 MHz, CDCl_3_): δ 141.9, 133.1, 131.2, 130.3, 129.0, 125.4, 122.5, 120.4, 77.7, 40.0; MS (ES+): 473 [M + Na]^+^; HRMS (ES+) calcd for C_20_H_18_Br_2_O_2_Na: 472.9552, found: 472.9565.

*4,5-Diallyl-4,5-dihydropyrene-4,5-diol* (**4d**): yield 100%, light yellow crystals, m.p. 137–139 °C; ^1^H-NMR (400 MHz, CDCl_3_): *δ* 7.80 (dd, 2H, 7.6, 1.2 Hz), 7.76 (s, 2H), 7.71 (d, 2H, 7.6 Hz), 7.62–7.58 (t, 2H, 7.6 Hz), 5.45–5.34 (m, 2H), 5.01–4.88 (m, 4H), 2.86–2.80 (m, 2H), 2.58 (br s, 2xOH), 2.37–2.32 (m, 2H); ^13^C-NMR (100 MHz, CDCl_3_): *δ* 139.1, 133.9, 131.1, 126.8, 126.7, 126.6, 125.4, 123.1, 119.4, 79.2, 40.8; HRMS (ES+) calcd for C_22_H_20_O_2_Na: 339.1361, found: 339.1359.

*4,5-Diallyl-9,10-dibromo-4,5-dihydropyrene-4,5-diol* (**4e**): yield 97%, colorless crystals, m.p. 178–182 °C; ^1^H-NMR (400 MHz, CDCl_3_): *δ* 8.34 (dd, 2H, 8.0, 1.2 Hz), 7.79 (dd, 2H, 7.2, 1.2 Hz), 7.69–7.65 (m, 2H), 5.40–5.30 (m, 2H), 5.02–4.88 (m, 4H), 2.84–2.78 (m, 2H), 2.72 (s, 2xOH), 2.32–2.26 (m, 2H); ^13^C-NMR (100 MHz, CDCl_3_): *δ* 139.4, 133.3, 130.8, 128.2, 128.1, 126.1, 125.4, 124.6, 119.9, 78.8, 40.7; Anal. calcd for C_22_H_18_Br_2_O_2_: C, 55.72; H, 3.83; found: C, 55.87; H, 3.56%.

*5,6-Diallyl-2,9-di-n-butyl-5,6-dihydro-1,10-phenanthroline-5,6-diol* (**4f**): yield 84%, white crystals (crystallizes as monohydrate), m.p. 121–123 °C; ^1^H-NMR (400 MHz, CDCl_3_ dry): *δ* 7.72 (d, 2H, 7.8 Hz), 7.19 (d, 2H, 7.8 Hz), 5.49–5.41 (m, 2H), 5.09–4.97 (m, 4H), 3.00–2.96 (m, 4H), 2.70–2.64 (m, 2H), 2.47 (s, 2xOH), 2.34–2.29 (m, 2H), 1.85–1.77 (m, 4H), 1.57 (2H, H_2_O), 1.47 (qt, 4H, 7.6, 7.6 Hz), 0.98 (t, 6H, 7.6 Hz); ^13^C-NMR (100 MHz, CDCl_3_): *δ* 162.6, 148.8, 134.3, 134.0, 133.2, 122.3, 120.3, 77.7, 40.5, 38.2, 31.8, 23.0, 14.2. HRMS (ES+) calcd for C_26_H_34_N_2_O_2_Na: 429.2518, found: 472.2520; Anal. calcd for C_26_H_36_N_2_O_3_: C, 73.55; H, 8.55; N, 6.60; found: C, 73.74; H, 8.30; N, 6.80%.

### 3.3. Pinacol Rearrangement of 9,10-Diallyl-9,10-dihydrophenanthrene-9,10-diol (***4a***)

The mixture of diol **4a** (1.932 g, 6.61 mmol), PTSA (1.287 g, 6.76 mmol) and trimethyl orthoformate (5.0 mL, 45.70 mmol) in toluene (10 mL) was heated to 110 °C with stirring under low flow of argon (bubbler) for 2 h. Next, the reaction mixture was concentrated via evaporation of volatiles under reduced pressure and the product **6** was isolated by column chromatography (*c*Hex:EA 10:1) in 99% yield as colorless to slightly yellow oil.

*10,10-Diallylphenanthren-9(10H)-one* (**6**): ^1^H-NMR (400 MHz, CDCl_3_): *δ* 8.12 (dd, 1H, 8.0, 1.6 Hz), 8.06–8.03 (m, 2H), 7.66 (td, 1H, 7.6, 1.6 Hz), 7.48–7.36 (m, 4H), 5.36–5.27 (m, 2H), 4.89–4.76 (m, 4H), 3.00–2.95 (m, 2H), 2.66–2.60 (m, 2H); ^13^C-NMR (100 MHz, CDCl_3_): *δ* 201.1, 140.2, 137.3, 134.6, 133.1, 130.6, 129.7, 128.9, 128.2, 128.0, 127.7, 127.1, 123.7, 123.0, 118.3, 55.3, 45.3; HRMS (ES+) calcd for C_20_H_18_ONa: 297.1255, found: 297.1252; Anal. calcd for C_20_H_18_O: C, 87.56; H, 6.61; found: C, 87.28; H, 6.39%.

### 3.4. Pinacol Rearrangement of 5,6-Diallyl-2,9-di-n-butyl-5,6-dihydro-1,10-phenanthroline-5,6-diol (***4f***)

To the camphorsulfonic acid (83 mg, 0.357 mmol) solution of diol **4f** (69 mg, 0.170 mmol) in toluene (3 mL) and trimethyl orthoformate (0.2 mL, 1.83 mmol) was added. The reaction mixture was heated to 100 °C with stirring under low flow of argon (bubbler) for 2.5 h. Next, the reaction mixture was cooled down and NaHCO_3_ (500 mg, 5.95 mmol) was added. The product **5** was isolated by column chromatography on Al_2_O_3_ neutral (*c*Hex:EA 4:1) in 62% yield as yellow crystals, m.p. 86–89 °C.

*6,6-Diallyl-2,9-dibutyl-1,10-phenanthrolin-5(6H)-one* (**5**): ^1^H-NMR (400 MHz, CDCl_3_): *δ* 8.28 (d, 1H, 8.0 Hz), 7.68 (d, 1H, 8.0 Hz), 7.30 (d, 1H, 8.0 Hz), 7.29 (d, 1H, 8.0 Hz), 5.28–5.18 (m, 2H), 4.84–4.72 (m, 4H), 3.07–2.94 (m, 6H), 2.61–2.56 (m, 2H), 1.88–1.79 (m, 4H), 1.52–1.43 (m, 4H), 1.48 (qt, 4H, 7.6, 7.6 Hz), 0.98 (td, 6H, 7.6, 1.6 Hz); ^13^C-NMR (100 MHz, CDCl_3_): δ 199.9, 169.9, 162.1, 153.7, 147.8, 135.5, 135.4, 134.7, 132.3, 125.1, 123.4, 123.4, 119.0, 55.2, 45.3, 39.2, 38.2, 31.5, 31.3, 22.9, 22.9, 14.1, 14.1; HRMS (ES+) calcd for C_26_H_32_N_2_ONa: 411.2412, found: 411.2396.

### 3.5. Ring Closing Metathesis of 10,10-Diallylphenanthren-9(10H)-one (***6***)

To the solution of ketone **6** (982 mg, 3.58 mmol) in DCM (5 mL) Umicore™ M2 catalyst (17 mg, 0.0179 mmol) under Ar at 22 °C was added. The reaction mixture was left overnight. Next, solvent was evaporated and product **7** was isolated by column chromatography (cHex:EA 25:1) in 99% yield as colorless crystals, m.p. 72–73 °C.

*10′H-Spiro[cyclopent[3]ene-1,9′-phenanthren]-10′-one* (**7**): ^1^H-NMR (400 MHz, CDCl_3_): *δ* 8.13–8.11 (m, 1H), 8.05–8.02 (m, 1H), 7.99–7.97 (m, 1H), 7.70–7.66 (m, 1H), 7.46–7.33 (m, 4H), 5.78 (s, 2H), 3.34–3.28 (m, 2H), 2.71–2.66 (m, 2H); ^13^C-NMR (100 MHz, CDCl_3_): *δ* 202.0, 145.3, 137.7, 134.5, 129.5, 129.4, 128.6, 128.5, 128.2, 128.1, 127.2, 126.5, 123.7, 123.2, 56.3, 47.6; HRMS (ES+) calcd for C_18_H_14_ONa: 269.0942, found: 269.0954.

### 3.6. One-Pot Reduction and Wagner-Meerwein Rearrangement of 10,10-Diallylphenanthren-9(10H)-one (***6***)

The solution of ketone **6** (58 mg, 0.211 mmol), MeSO_3_H (0.10 mL, 1.54 mmol) and Et_3_SiH (0.10 mL, 0.626 mmol) in DCM (0.5 mL) was stirred at 22 °C under argon for 30 min. Next, the reaction was quenched with NaHCO_3_ (500 mg). Precipitate was filtered off, washed with DCM, and the product **9** was isolated by column chromatography (*c*Hex:DCM 10:1) in 81% yield as colorless needles, m.p. 108–109 °C.

*9,10-Diallylphenanthrene* (**9**): ^1^H-NMR (400 MHz, CDCl_3_): *δ* 8.75–8.72 (m, 2H), 8.10–8.07 (m, 2H), 7.63–7.60 (m, 4H), 6.19–6.12 (m, 2H) 5.09–4.93 (m, 4H), 3.93–3.90 (m, 4H); ^13^C-NMR (100 MHz, CDCl_3_): *δ* 136.3, 132.0, 131.5, 130.2, 126.8, 125.9, 125.3, 123.0, 116.1, 33.4; MS (EI+): m/z (%) = 258 (M^+^, 45), 230 (34), 228 (27), 218 (61), 216 (100), 203 (57), 114 (30); Anal. calcd for C_20_H_18_: C, 92.98; H, 7.02; found: C, 92.64; H, 6.81%.

### 3.7. One-Pot Reduction and Wagner-Meerwein Rearrangement of 10′H-Spiro[cyclopent[3]ene-1,9′-phenanthren]-10′-one (***7***)

To the solution of ketone **7** (123 mg, 0.50 mmol) in CF_3_COOH (0.5 mL) Et_3_SiH (0.10 mL, 0.643 mmol) at 22 °C was added. Formation of white precipitate was observed. After 5 min, Et_2_O (2 mL) was added and precipitate was filtered off and washed with Et_2_O. The product **8** was crystallized from hot EtOH. Yield 70%, colorless needles, m.p. 200–202 °C (lit. [36] 203–204 °C).

*1,4-Dihydro-triphenylene* (**8**): ^1^H-NMR (400 MHz, DMSO-*d_6_*, 90 °C): *δ* 8.83–8.80 (m, 2H), 8.08–8.05 (m, 2H), 7.70–7.66 (m, 4H), 6.14 (s, 2H), 3.77 (s, 4H); ^13^C-NMR (100 MHz, DMSO-*d_6_*, 90 °C): *δ* 130.0, 128.7, 126.3, 126.1, 125.5, 123.0, 122.9, 122.3, 26.6.

### 3.8. Direct Dehydroxylation of 9,10-Diallyl-9,10-dihydrophenanthrene-9,10-diol (***4a***) to 9,10-Diallylphenanthrene (***9***)

To a solution of diol **4a** (285 mg, 0.975 mmol) in DCM (2 mL), a solution of B(C_6_F_5_)_3_ (10 mg, 0.0195 mmol) and Et_3_SiH (0.5 mL, 3.13 mmol) in DCM (0.5 mL) under argon at 22 °C was added. The reaction mixture was stirred for 1 h, and next the product **9** was isolated by column chromatography (*n*Hex). Yield 81%.

### 3.9. Ring Closing Metathesis of 9,10-Diallylphenanthrene (***9***)

To the solution of compound **9** (22 mg, 0.0852 mmol) in CDCl_3_ (1 mL), Umicore™ M2 catalyst (2.0 mg, 0.00211 mmol) under Ar at 22 °C was added. The reaction mixture was left overnight. Next, ^1^H-NMR spectrum was recorded, showing full conversion of substrate to 1,4-dihydro-triphenylene (**8**). The product **8** was isolated by column chromatography (*c*Hex:DCM 1:1) in 95% yield.

### 3.10. X-ray Diffraction Data

Single-crystal X-ray diffraction data for **4c** were collected on a Supernova Dual Source diffractometer, equipped with an Atlas detector using CuK_α_ radiation (λ = 1.54178 Å). The CrysAlisPro software system was used for the data collection, cell refinement, and data reduction [37]. Empirical absorption correction was applied. The structure was solved using the direct method with SHELXS-97 and refined by full-matrix least-squares methods based on F^2^ with SHELXL-97 [38]. All non-hydrogen atoms were refined anisotropically. Hydrogen atoms, except those coming from -OH, were positioned geometrically with C-H = 0.99–0.95 Å and refined as riding with U_iso_(H) = 1.2 Ueq (C). The O-H hydrogen atoms were located in a difference map and refined. The program MERCURY [39] was used to prepare the molecular graphic.

Crystallographic data for **4c**: (C_20_H_18_Br_2_O_2_), *M* = 450.16, colorless block, 0.14 mm × 0.12 mm × 0.09 mm, triclinic, space group *P*1(No. 2), *a* = 7.0952(3), *b* = 11.6593(6), *c* = 12.6457(7) Å, *α* = 116.795(5), *β* = 94.011(4), *γ* = 104.067(4)°, *V* = 886.11(8) Å^3^, *Z* = 2, *D*_c_ = 1.687 g/cm^−3^, *F*_000_ = 448, *T* = 100(2) K, 2*θ*_max_ = 153.0°, 6230 reflections collected, 3628 unique (R_int_ = 0.0210). Final *GooF* = 1.058, *R*_1_ = 0.0268, *wR*_2_ = 0.0699, *R* indices based on 3332 reflections with I > 2σ(I) (refinement on *F*^2^), 225 parameters, 0 restraints. Lp and absorption corrections applied, *μ* = 5.872 mm^−1^.

CCDC-1853046 (for **4c**) contains the supplementary crystallographic data for this paper. These data can be obtained free of charge from The Cambridge Crystallographic Data Centre via www.ccdc.cam.ac.uk/structures.

## 4. Conclusions

A new strategy for the synthesis of polycyclic aromatic systems, based on a four-step sequence starting from polycyclic orthoquinones, has been proposed. The first stage, a method for the addition of allyl substituents to carbonyl groups in orthoquinones, has been well optimized and leads to diols in excellent yields. Studies to confirm the general applicability and limitations for the full sequence of the proposed strategy, with examples containing electron-donating (Me, OMe) and electron-withdrawing (CF_3_, CN) substituents, are being undertaken, and will be communicated in due course.

## Figures and Tables

**Table 1:** Synthesis of diallyl diols ^a^_._

Entry	Substrate	Product	Yield (%) ^b^
1	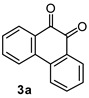	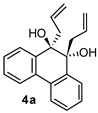	93
2	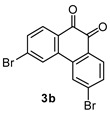	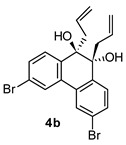	85
3	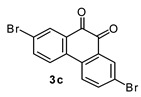	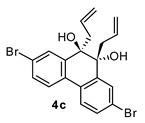	94
4	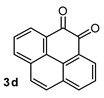	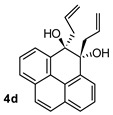	100
5	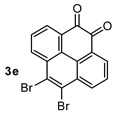	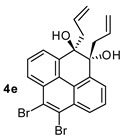	97
6	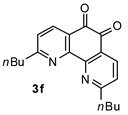	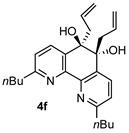	84

^a^ Reaction conditions: boron compound (**1**) (1.49 mmol), allylmagnesium bromide (12 mmol), quinone (**3**) (3 mmol), Et_2_O (20 mL), 0 °C. ^b^ Isolated yield.

## Supplementary Materials

Copies of the NMR spectra are available online.
