# Supplementary material for: Allylation of Orthoquinones Towards Annulated Polycyclic Aromatic Systems
Source: Molecules. 2018 Aug 15;23(8):2043. doi: 10.3390/molecules23082043 (PMC6222356; doi:10.3390/molecules23082043)

Supporting Information

Allylation of Orthoquinones Towards Annulated Polycyclic Aromatic Systems

Mariusz Kędziorek [1,†,*] and Liliana Dobrzańska [2]

[1] Faculty of Chemistry, University of Warsaw, Żwirki i Wigury 101, 02-089 Warsaw, Poland

[2] Faculty of Chemistry, Nicolaus Copernicus University in Toruń, Gagarina 7, 87-100 Toruń, Poland; lianger@umk.pl

* Correspondence: m.kedziorek@cent.uw.edu.pl; Tel.: +48-22-55-43644

† Present address: Centre of New Technologies, University of Warsaw, Banacha 2c, 02 097 Warsaw, Poland

Solvent: cdcl3

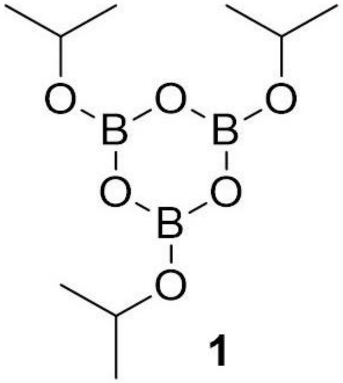

1

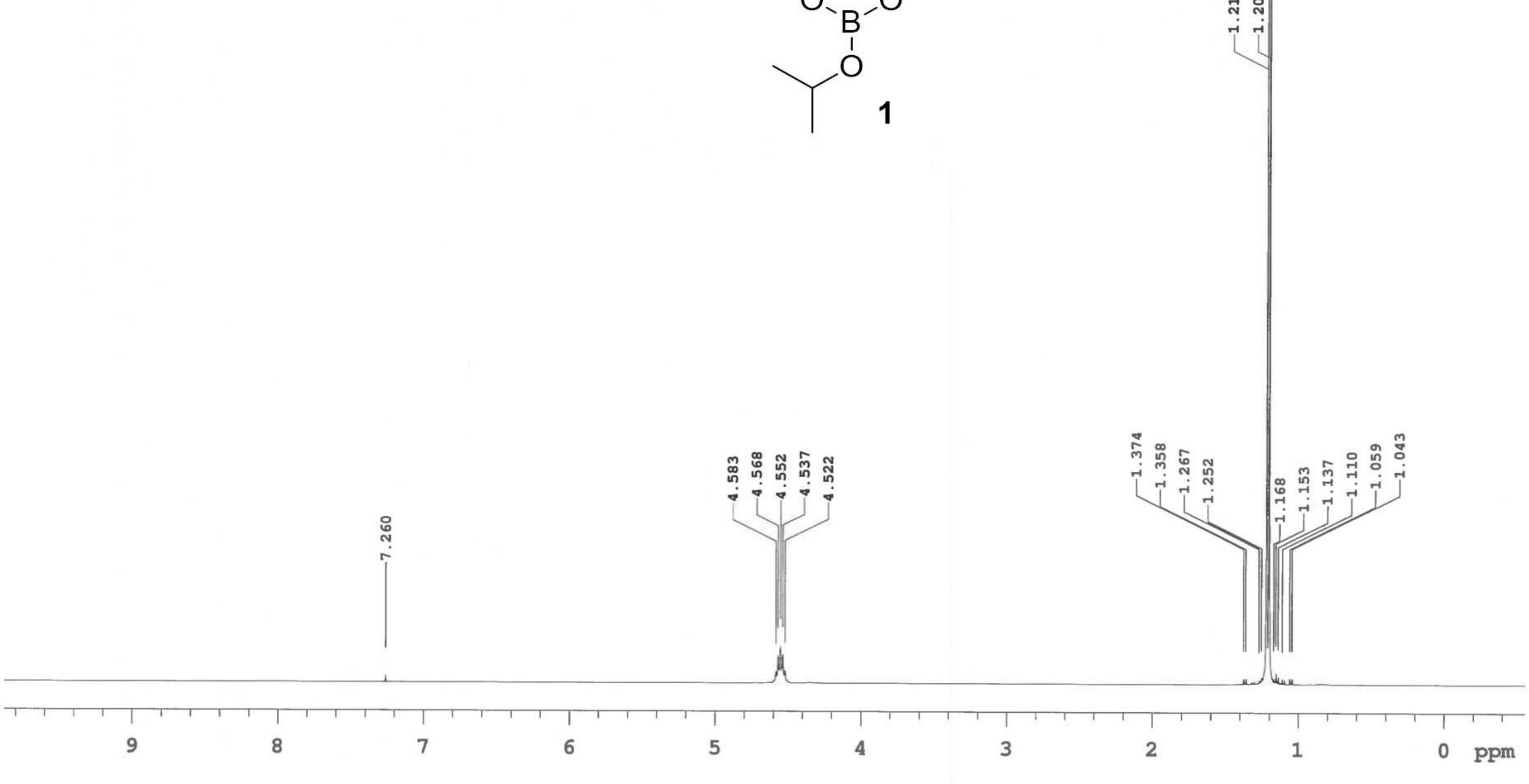

Solvent: cdcl3

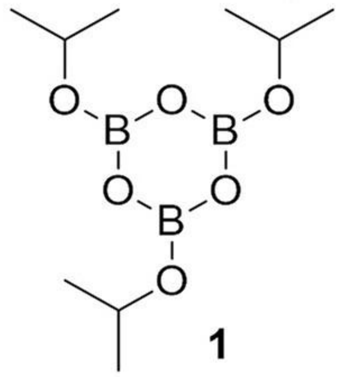

1

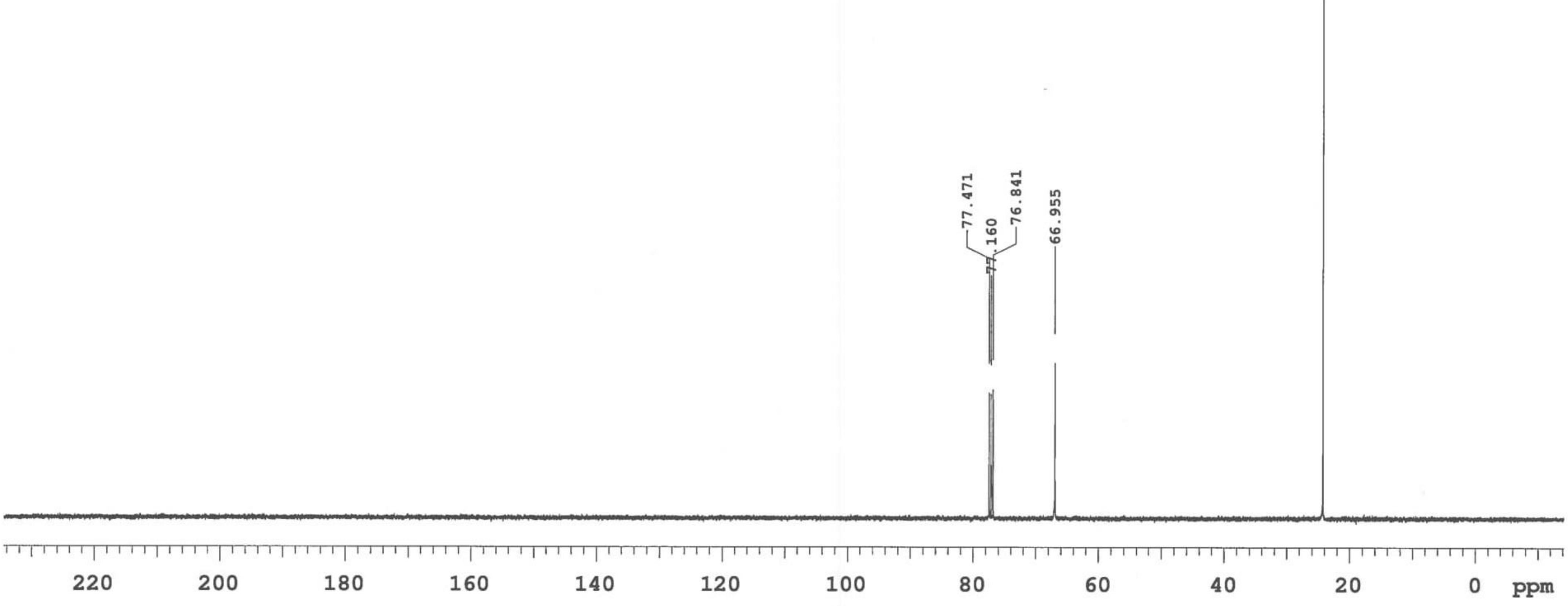

Solvent: cdcl3

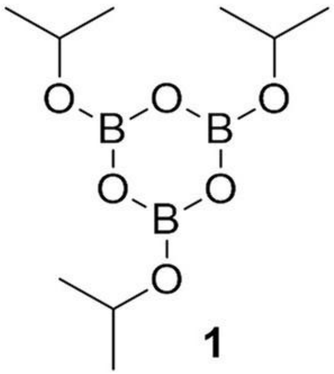

1

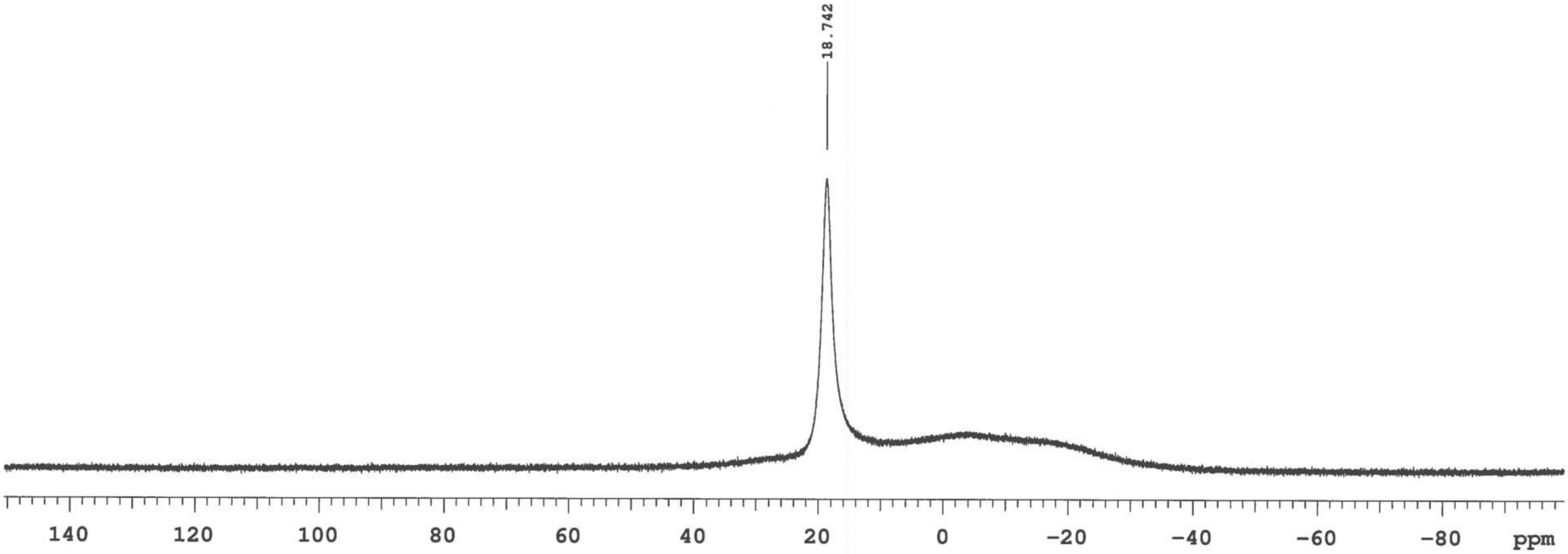

Solvent: cdcl3

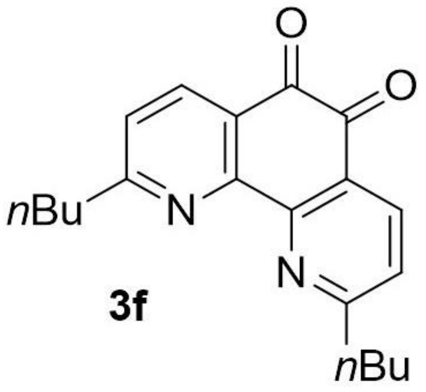

3f

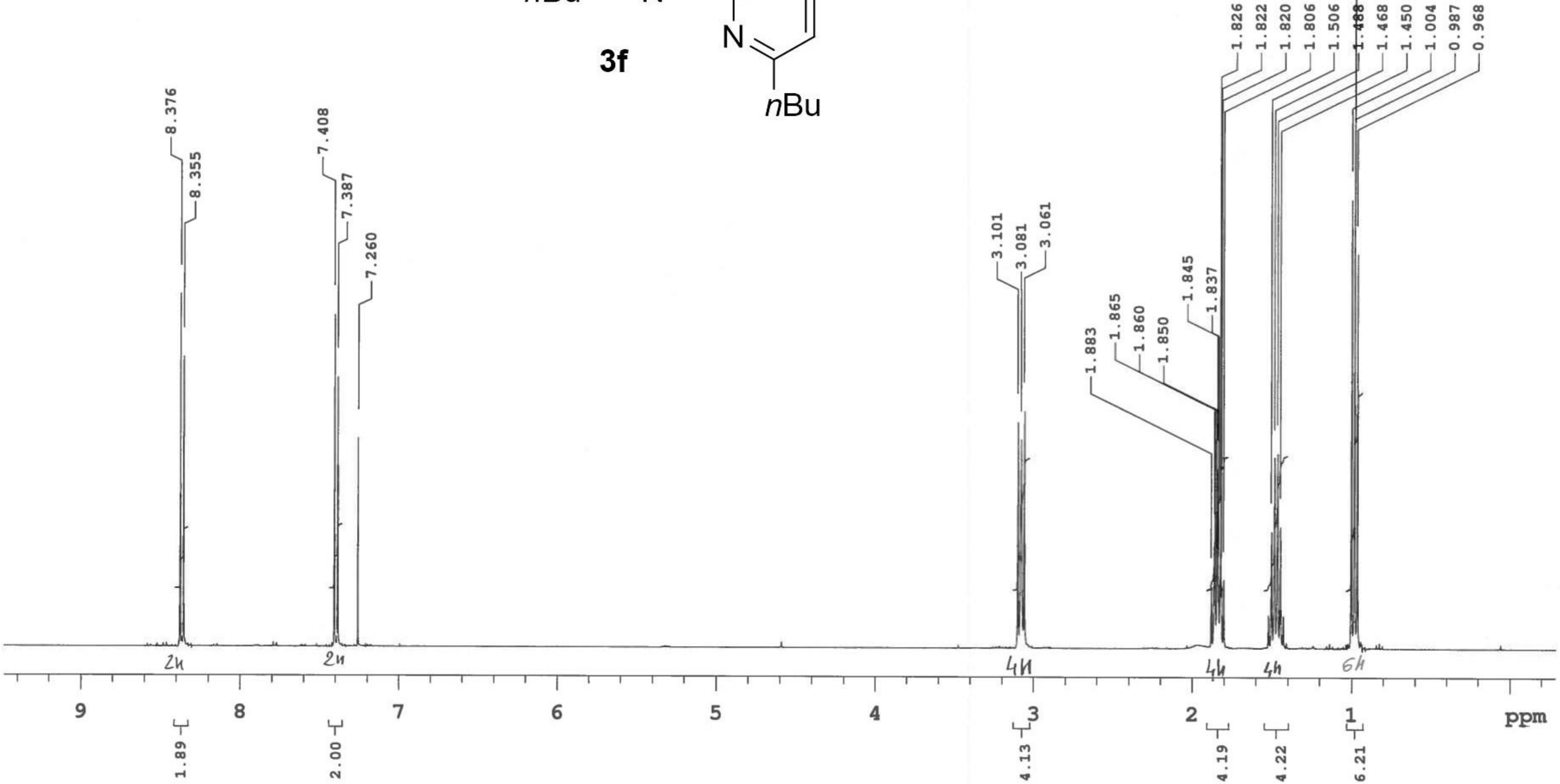

Solvent: cdcl3

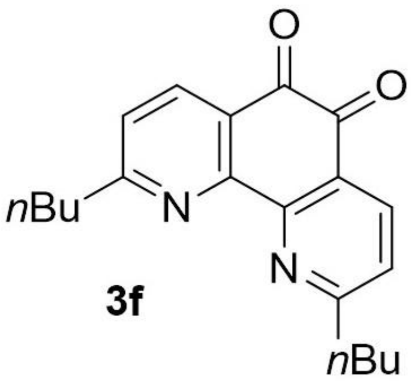

3f

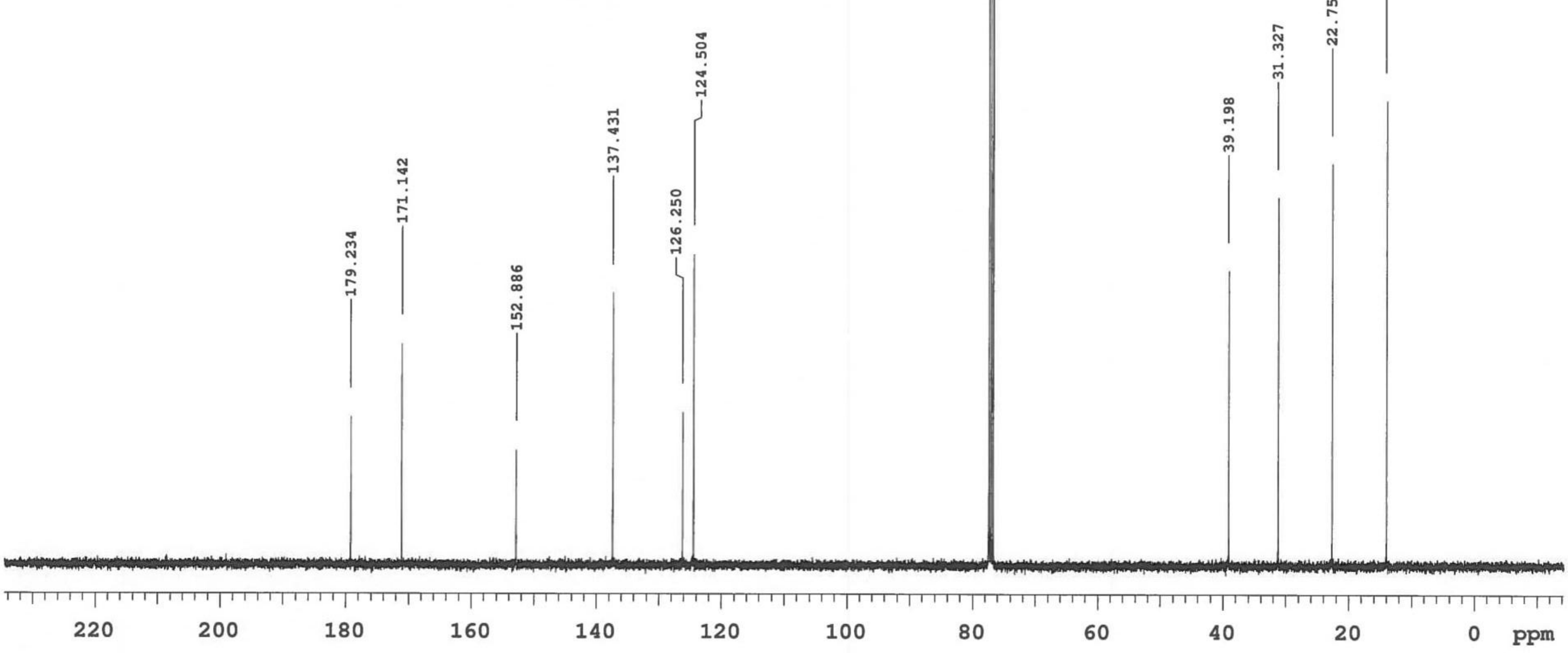

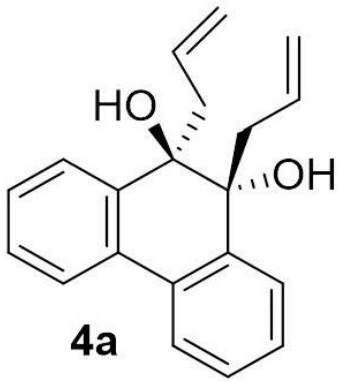

4a

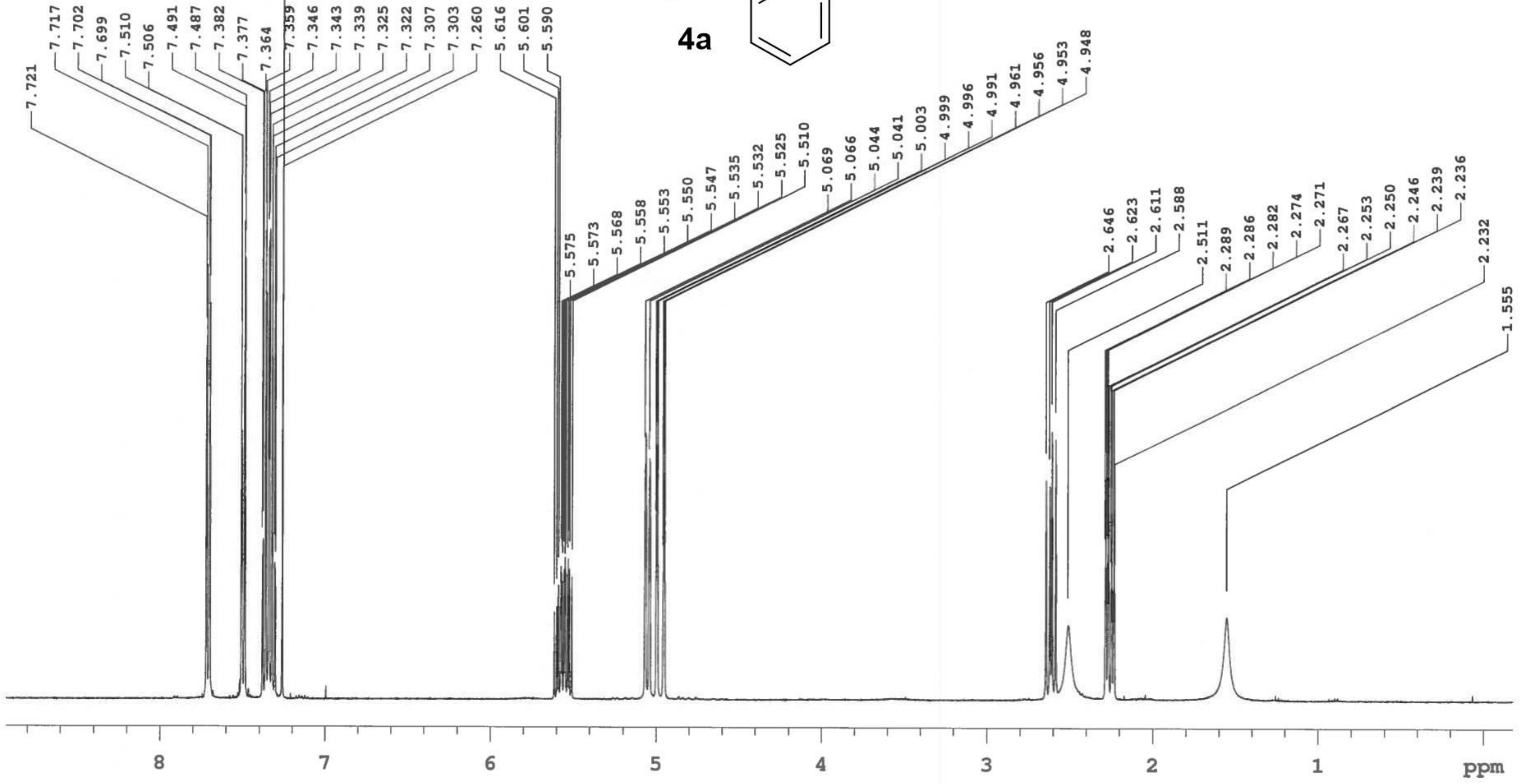
Solvent: cdcl3
7.721
7.717
7.702
7.699
7.510
7.506
7.491
7.487
7.382
7.377
7.364
7.359
7.346
7.343
7.339
7.325
7.322
7.307
7.303
7.260
5.616
5.601
5.590
5.575
5.573
5.568
5.558
5.553
5.550
5.547
5.535
5.532
5.525
5.510
5.069
5.066
5.044
5.041
5.003
4.999
4.996
4.991
4.961
4.956
4.953
4.948
2.646
2.623
2.611
2.588
2.511
2.289
2.286
2.282
2.274
2.271
2.267
2.253
2.250
2.246
2.239
2.236
2.232
1.555
8
7
6
5
4
3
2
1
ppm

Solvent: cdcl3

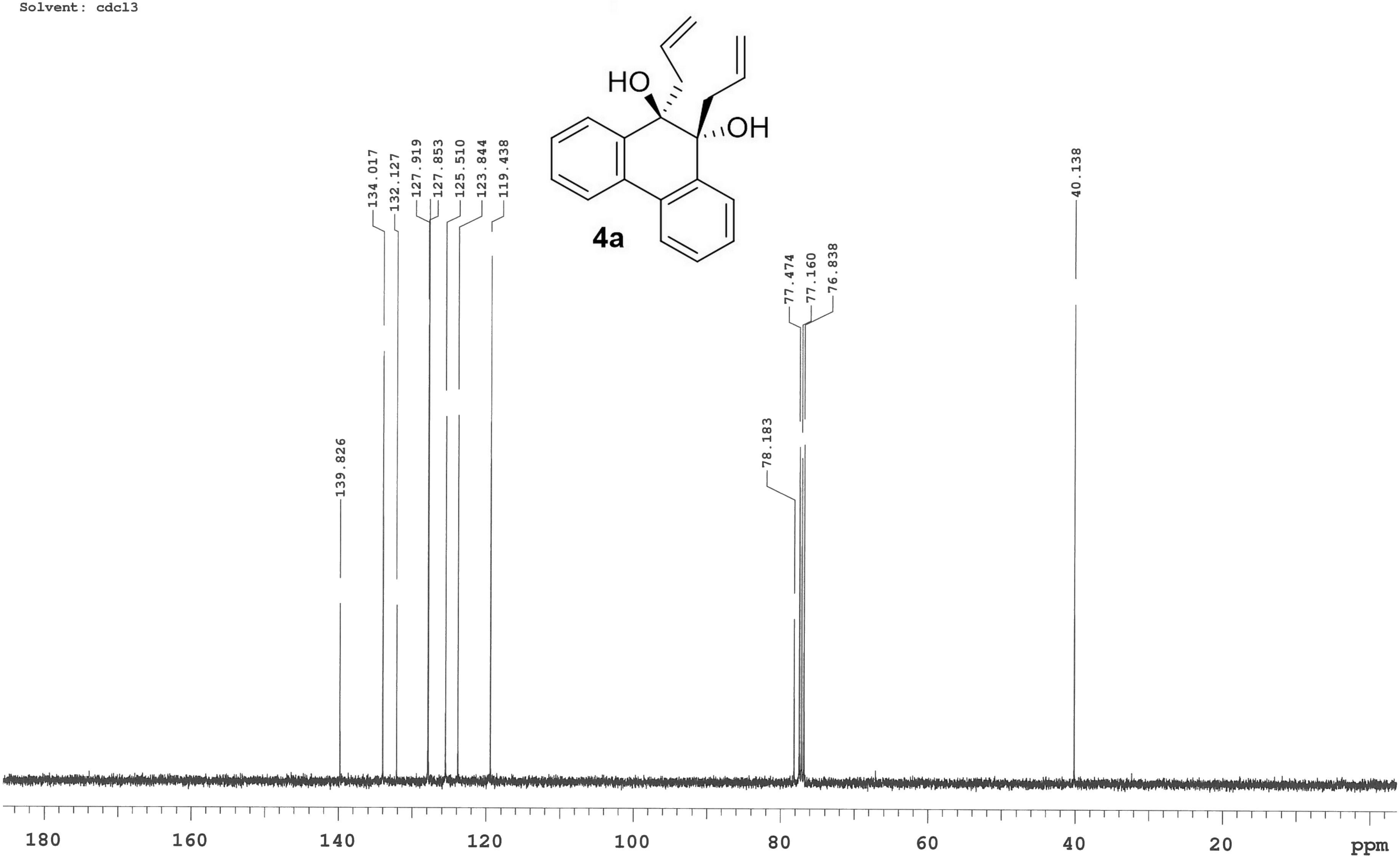

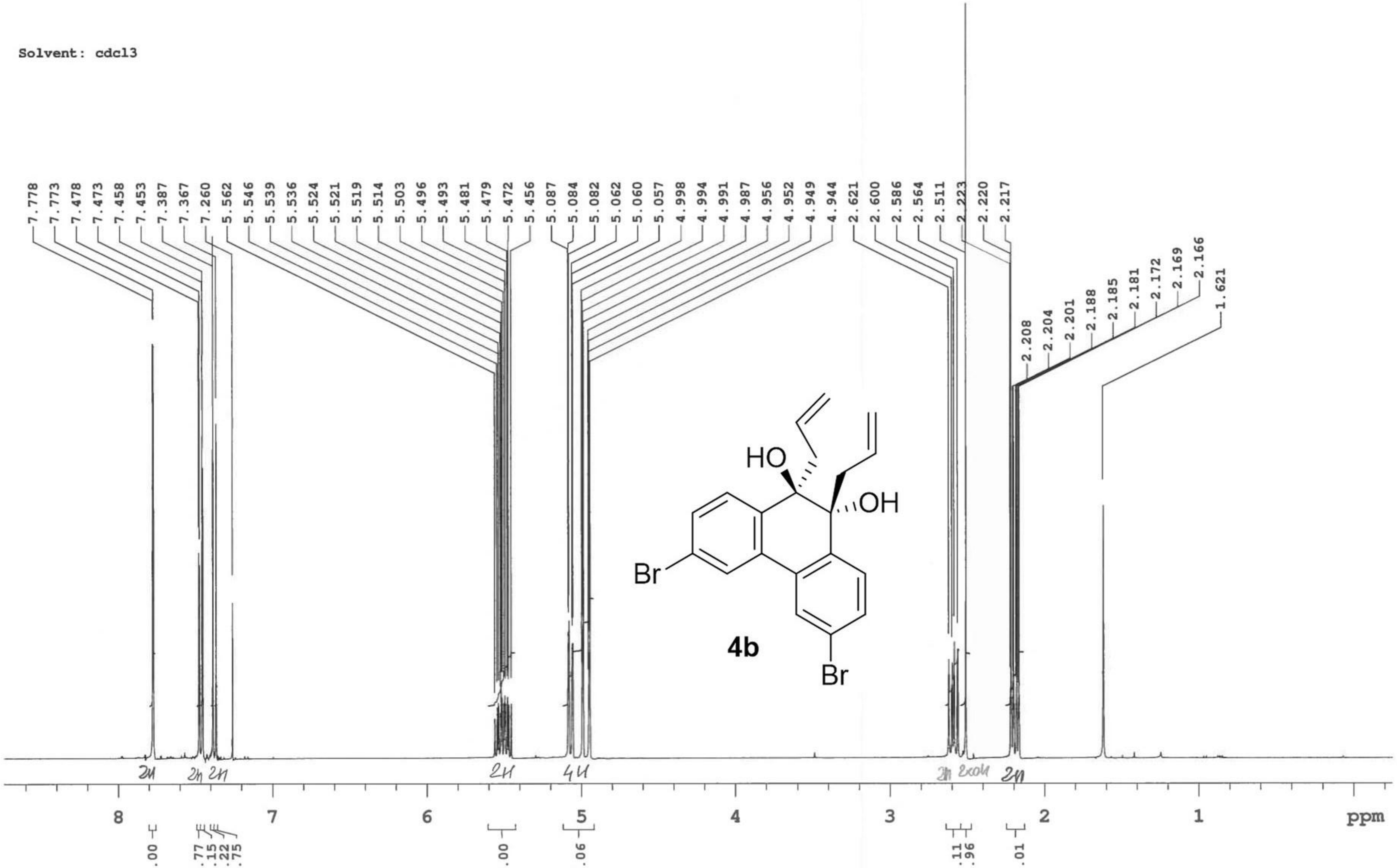
Solvent: cdcl3
7.778 7.773 7.478 7.473 7.458 7.453 7.387 7.367 7.260 5.562 5.546 5.539 5.536 5.524 5.521 5.519 5.514 5.503 5.496 5.493 5.481 5.479 5.472 5.456 5.087 5.084 5.082 5.062 5.060 5.057 4.998 4.994 4.991 4.987 4.956 4.952 4.949 4.944 2.621 2.600 2.586 2.564 2.511 2.223 2.220 2.217 2.208 2.204 2.201 2.188 2.185 2.181 2.172 2.169 2.166 1.621
HO
OH
Br
Br
4b
8
7
6
5
4
3
2
1
ppm
2.00
0.77
1.15
1.22
0.75
2.00
4.06
2.11
1.96
2.01

Solvent: cdcl3

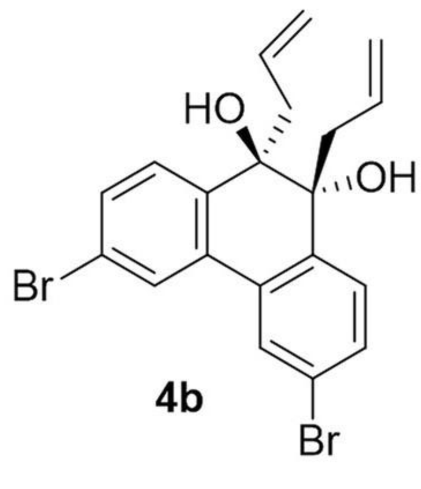

4b

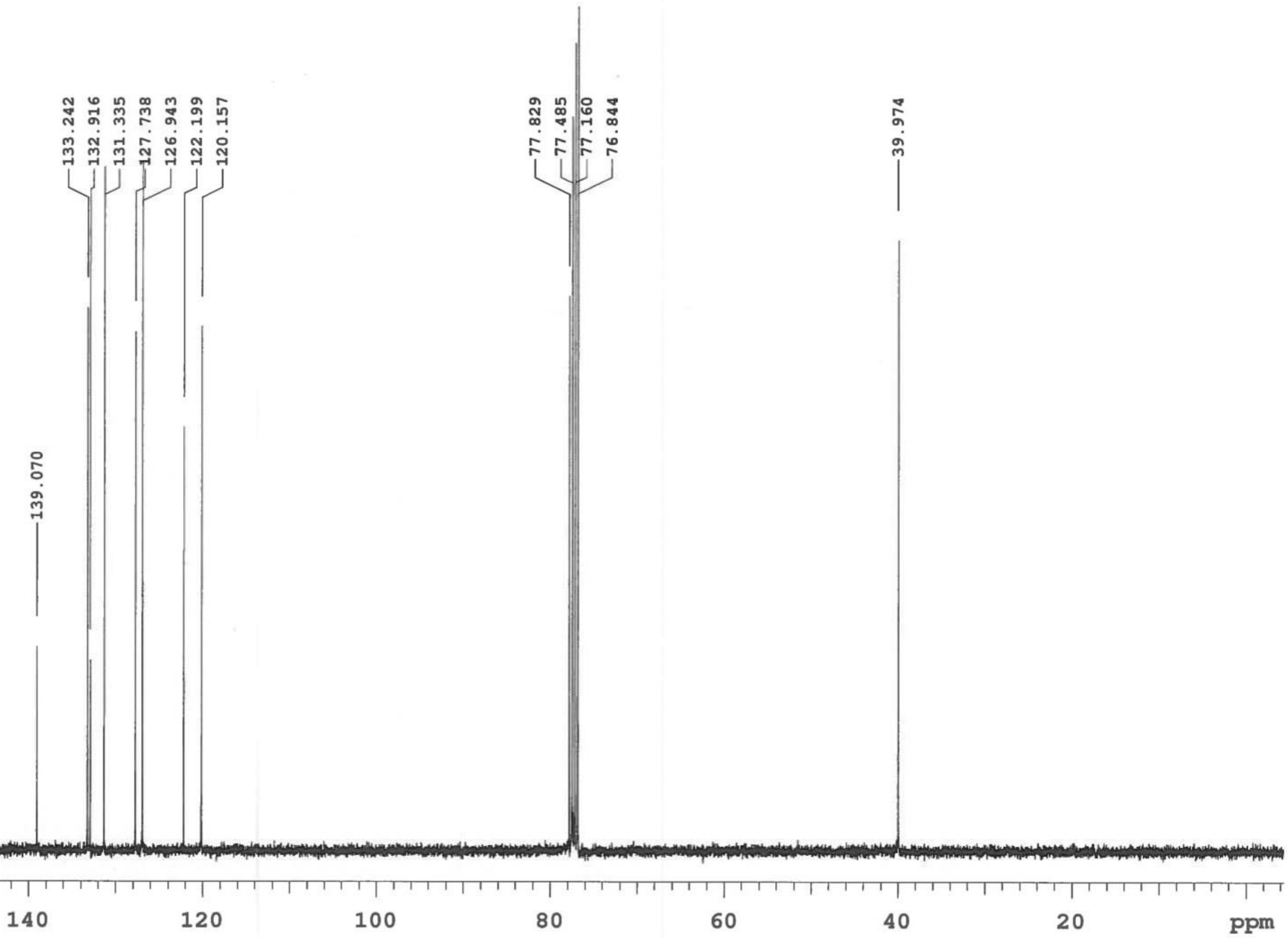

Solvent: cdcl3

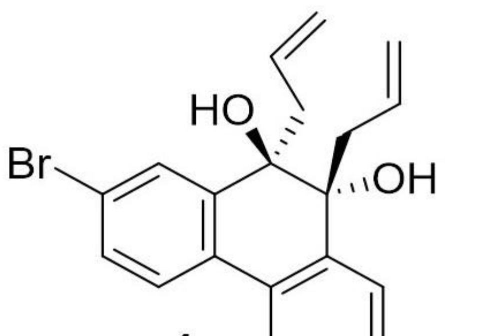

4c

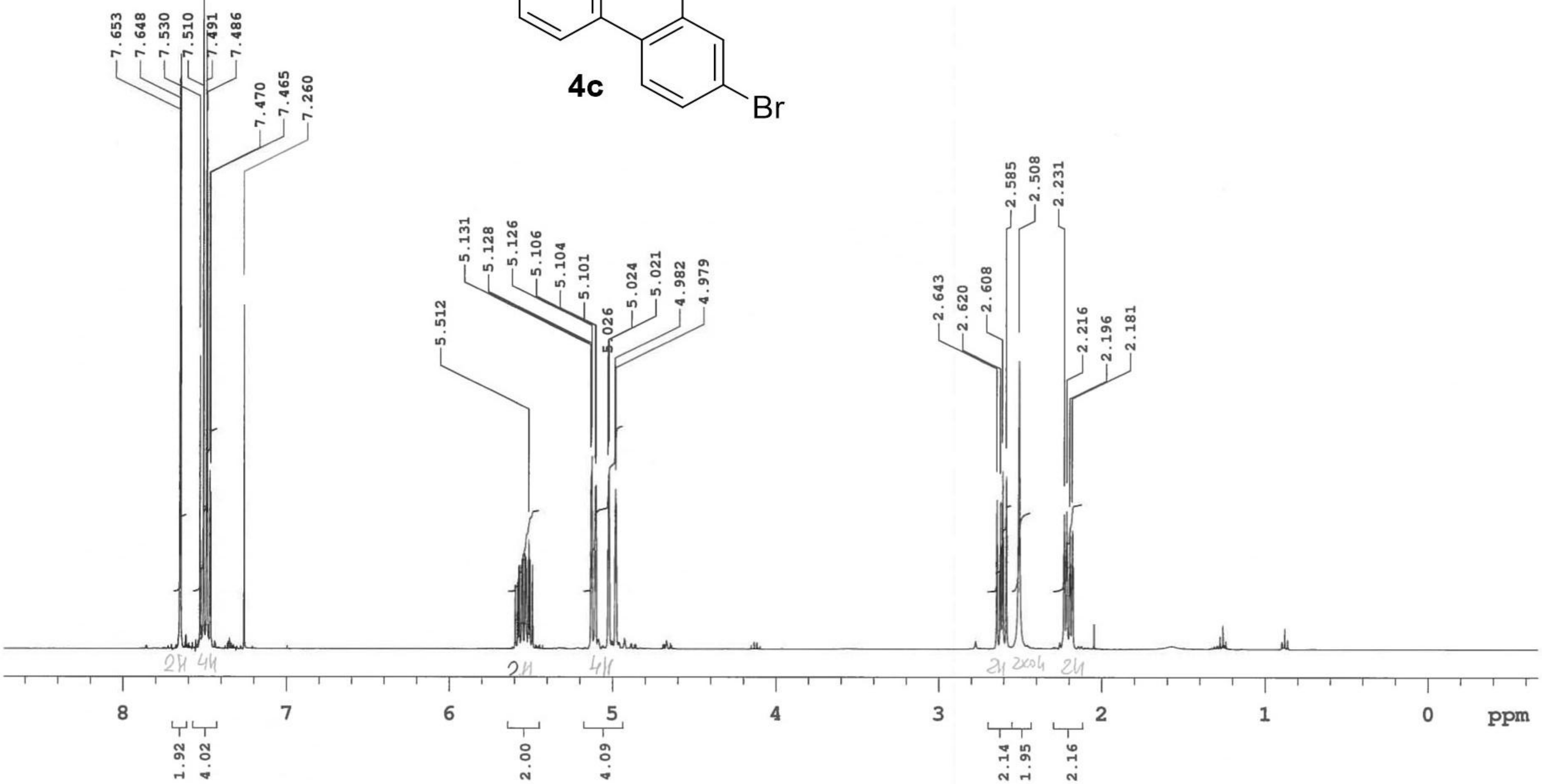

Solvent: cdcl3

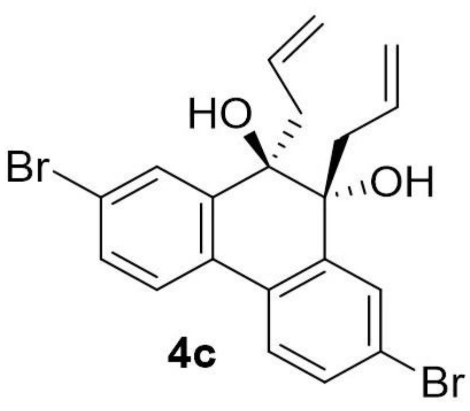

4c

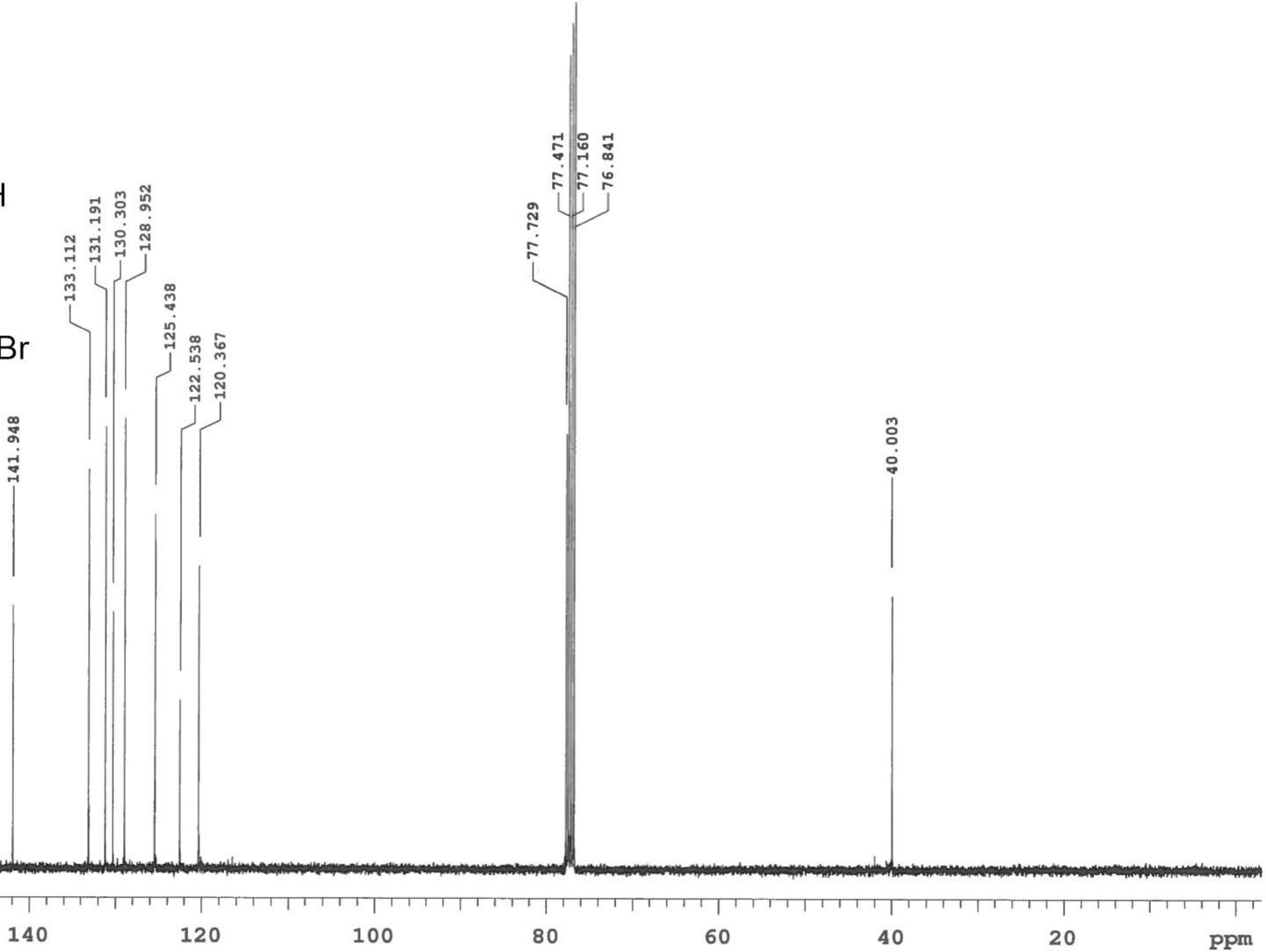

Solvent: cdcl3

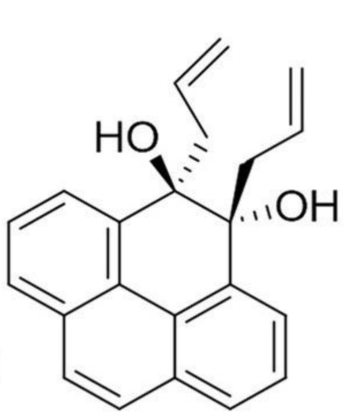

4d

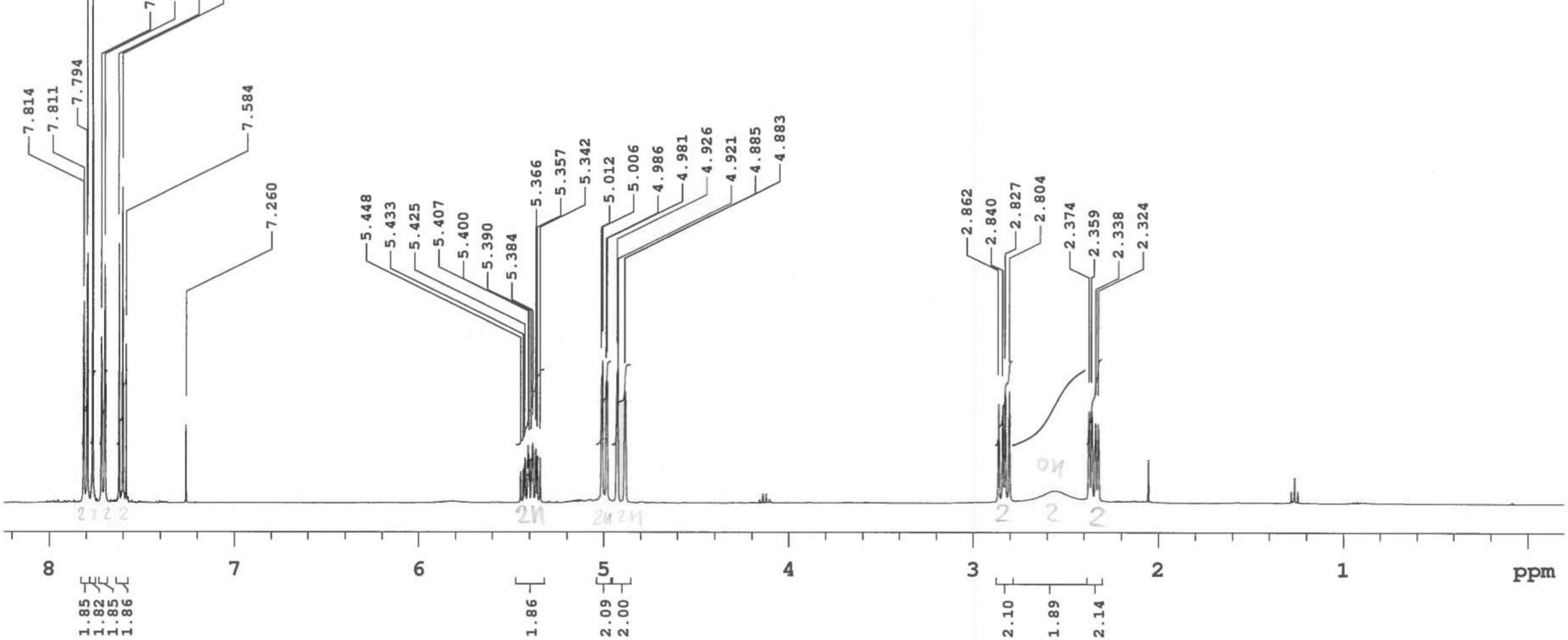

Solvent: cdcl3

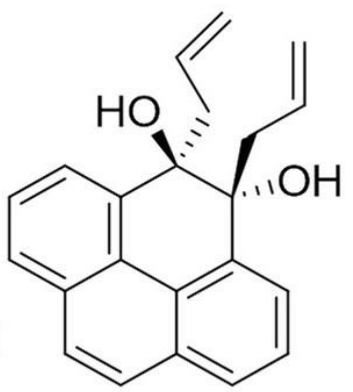

4d

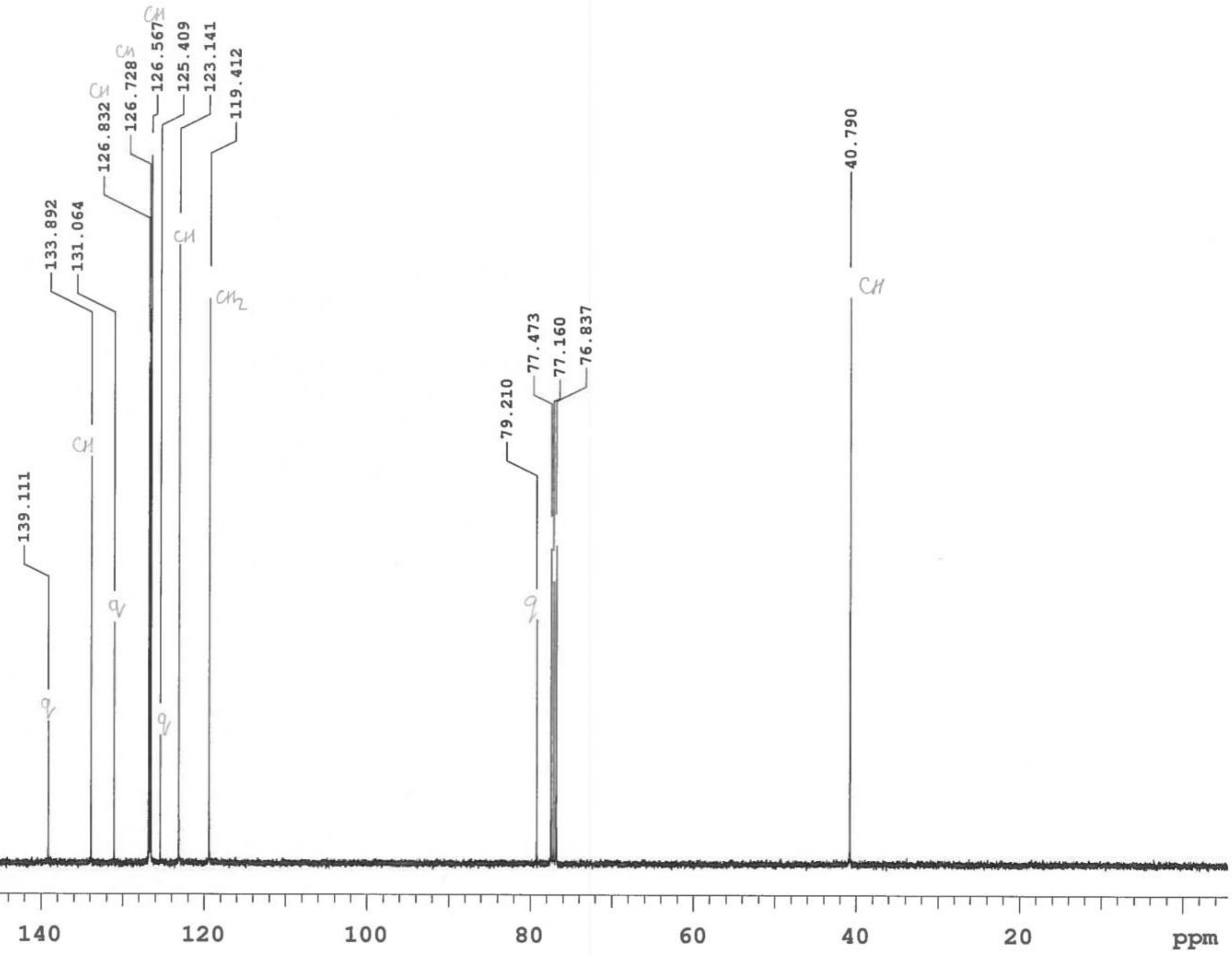

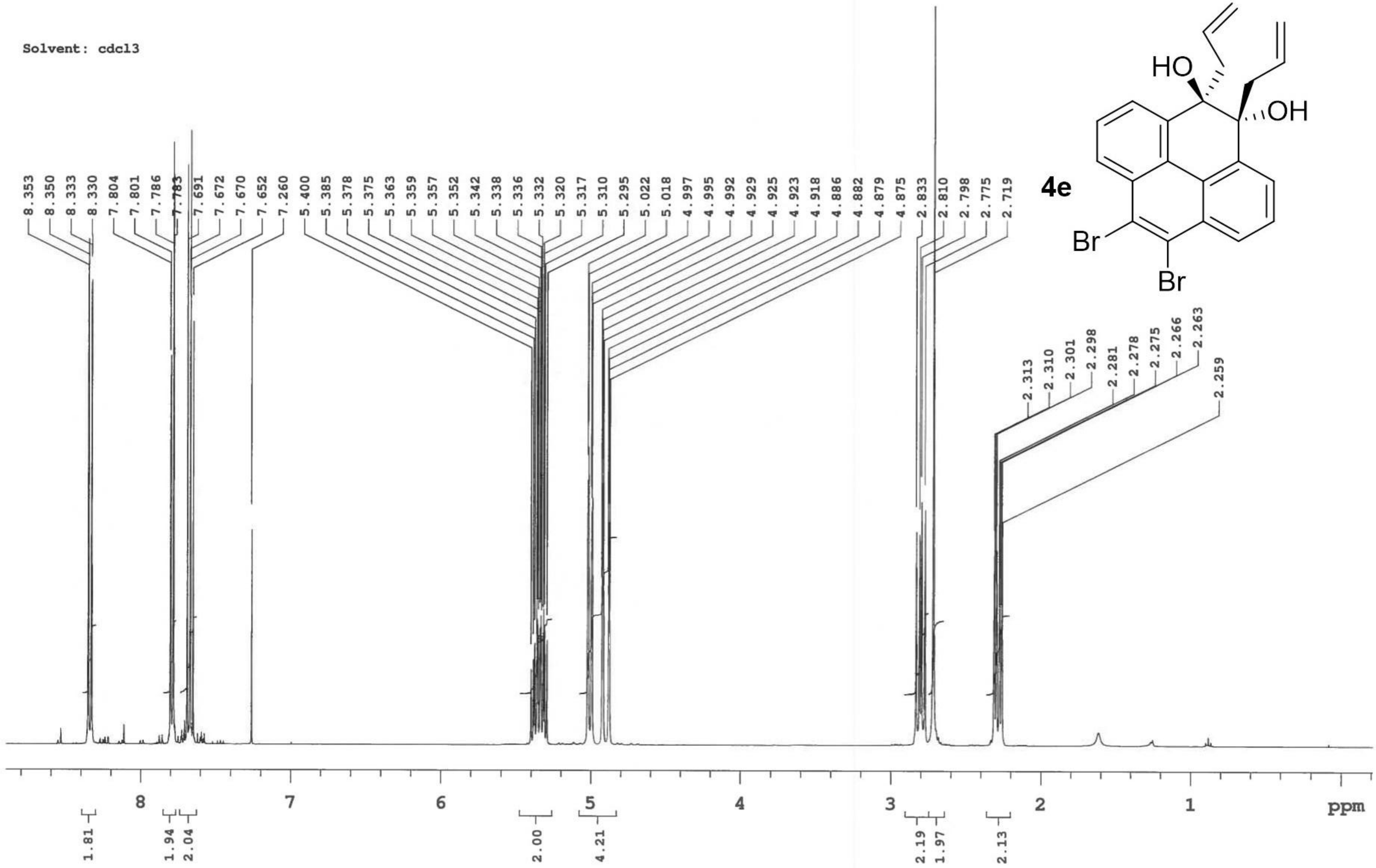
Solvent: cdcl3
8.353
8.350
8.333
8.330
7.804
7.801
7.786
7.783
7.691
7.672
7.670
7.652
7.260
5.400
5.385
5.378
5.375
5.363
5.359
5.357
5.352
5.342
5.338
5.336
5.332
5.320
5.317
5.310
5.295
5.022
5.018
4.997
4.995
4.992
4.929
4.925
4.923
4.918
4.886
4.882
4.879
4.875
2.833
2.810
2.798
2.775
2.719
2.313
2.310
2.301
2.298
2.281
2.278
2.275
2.266
2.263
2.259
8
7
6
5
4
3
2
1
ppm
1.81
1.94
2.04
2.00
4.21
2.19
1.97
2.13
HO
OH
Br
Br

4e

Solvent: cdcl3

4e

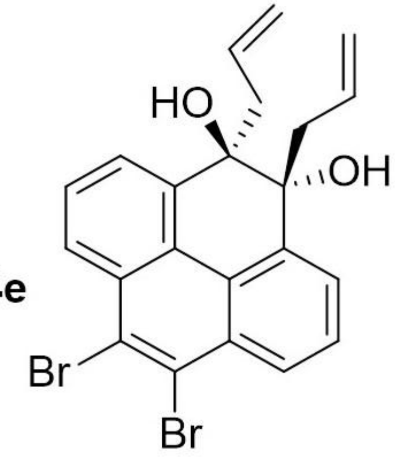

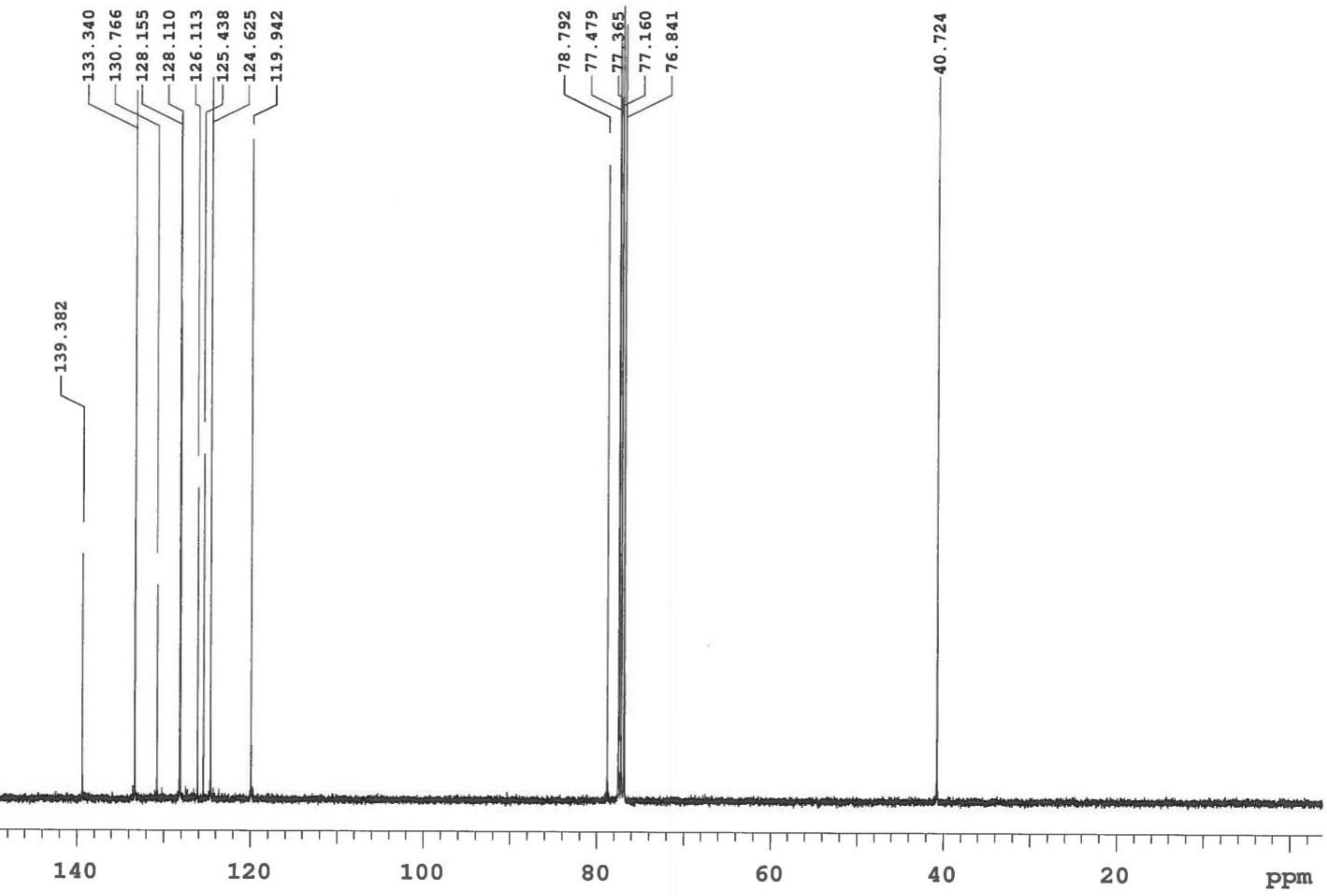

Solvent: cdcl3-dry

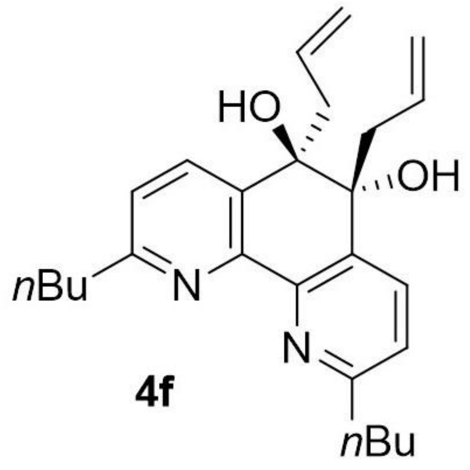

4f

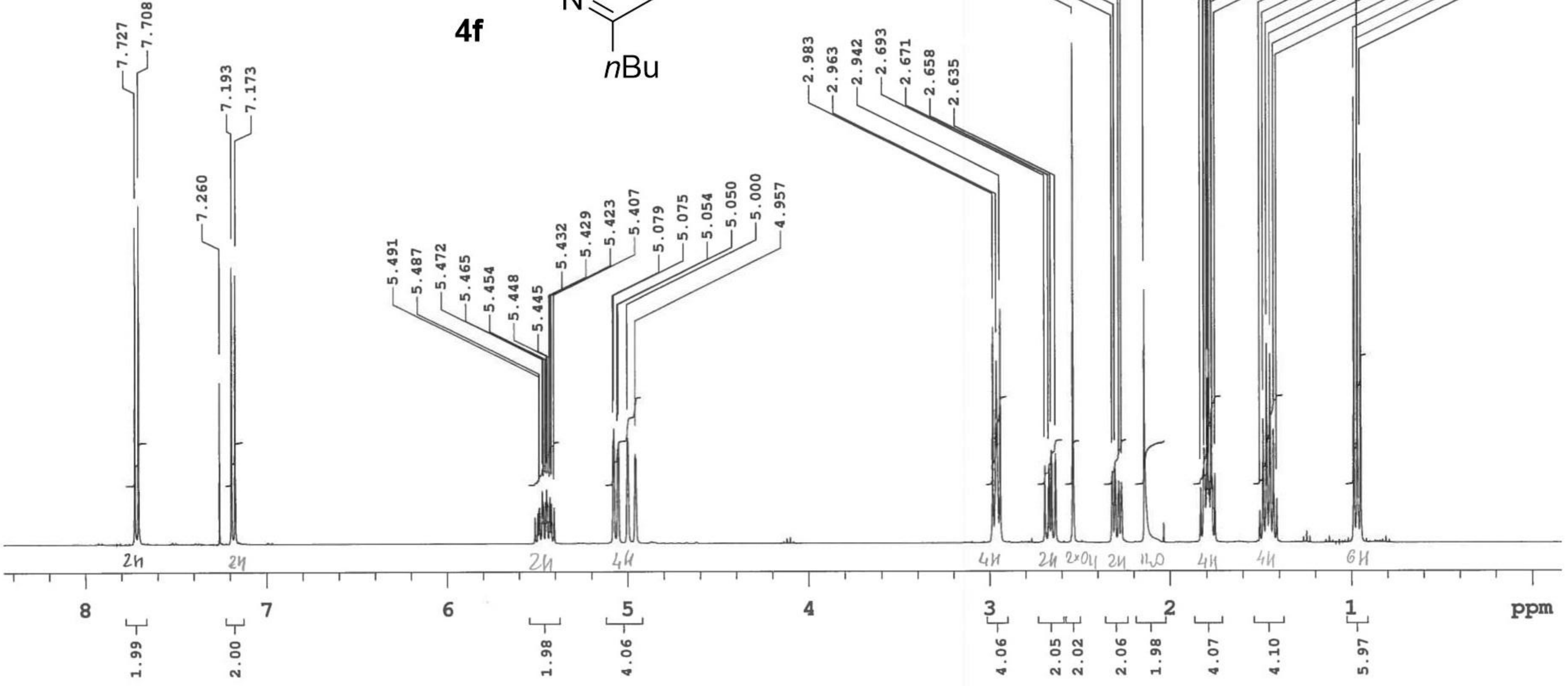

Solvent: cdcl3-dry

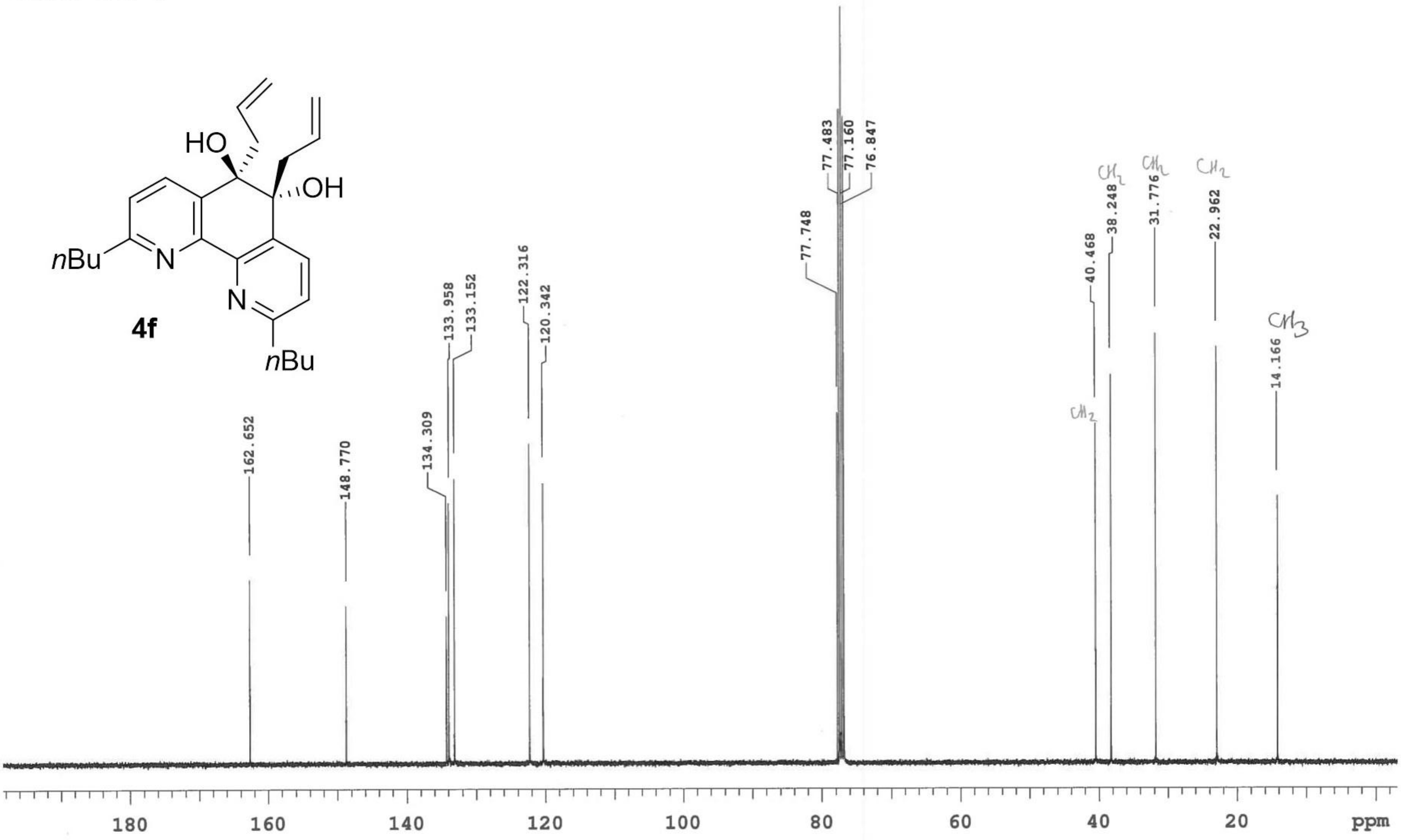

Solvent: cdcl3

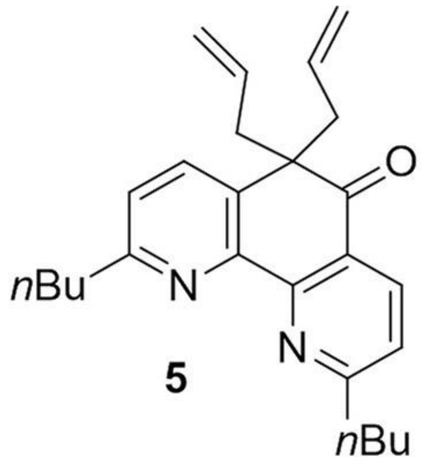

5

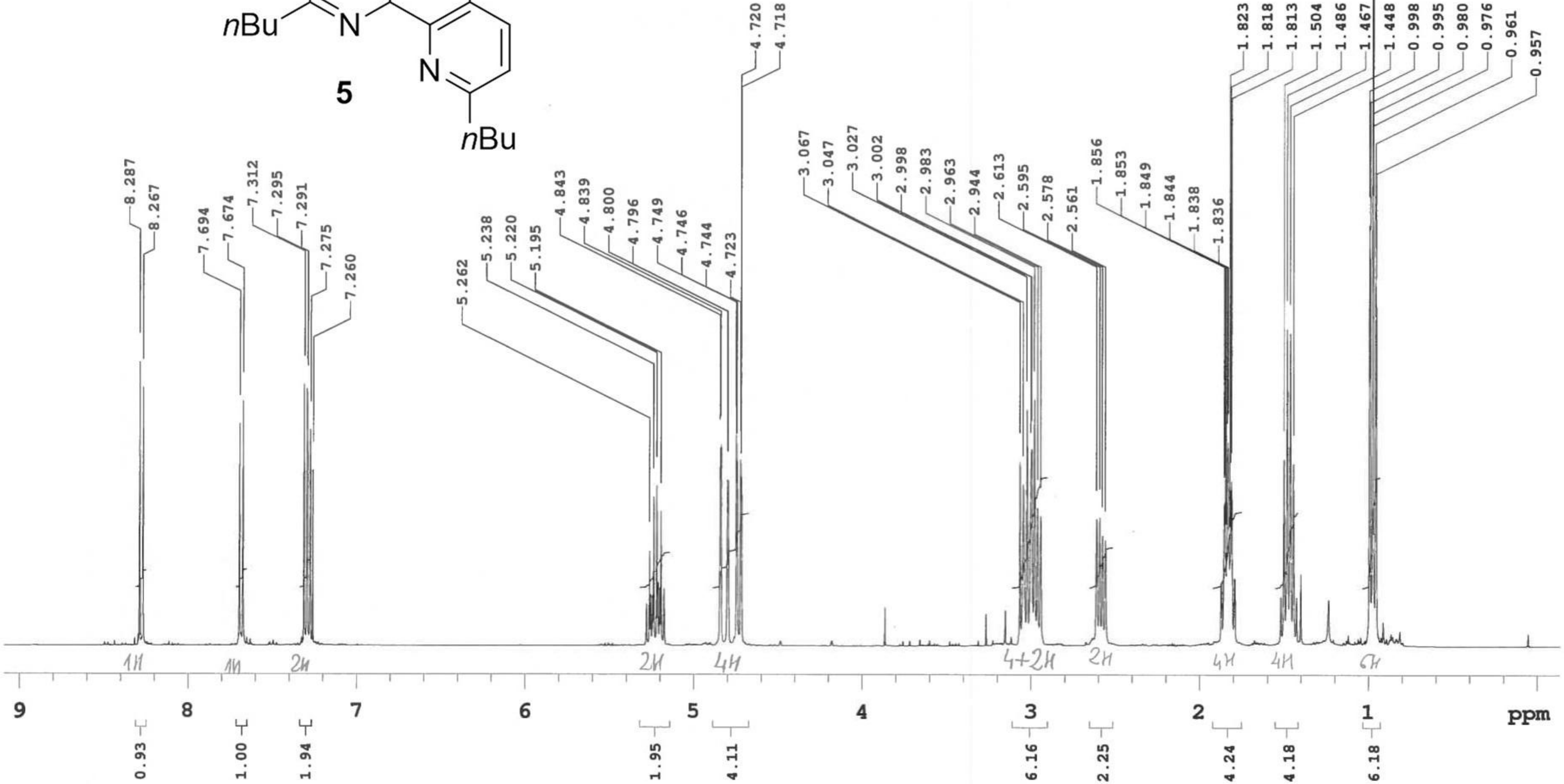

Solvent: cdcl3

5

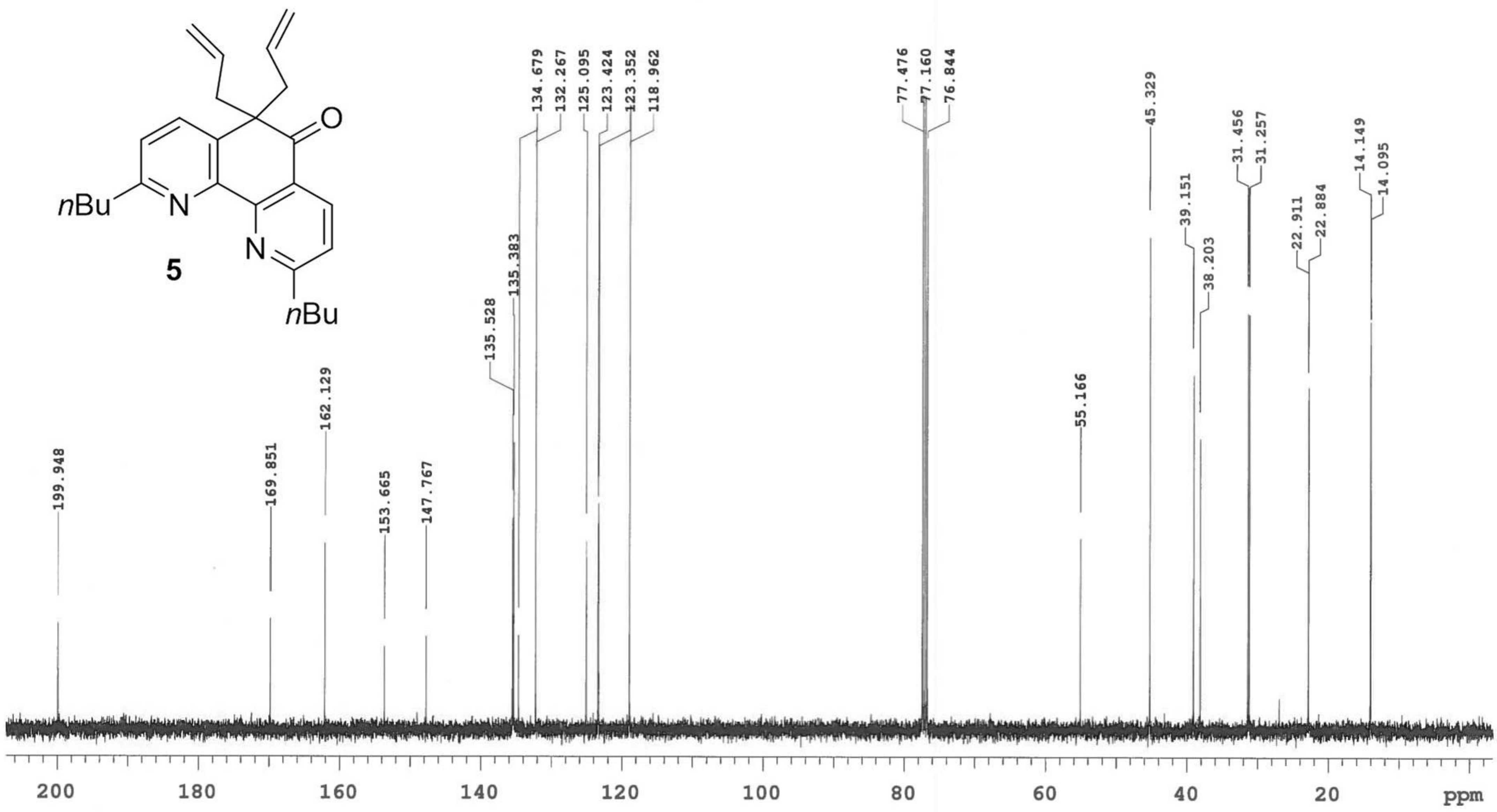

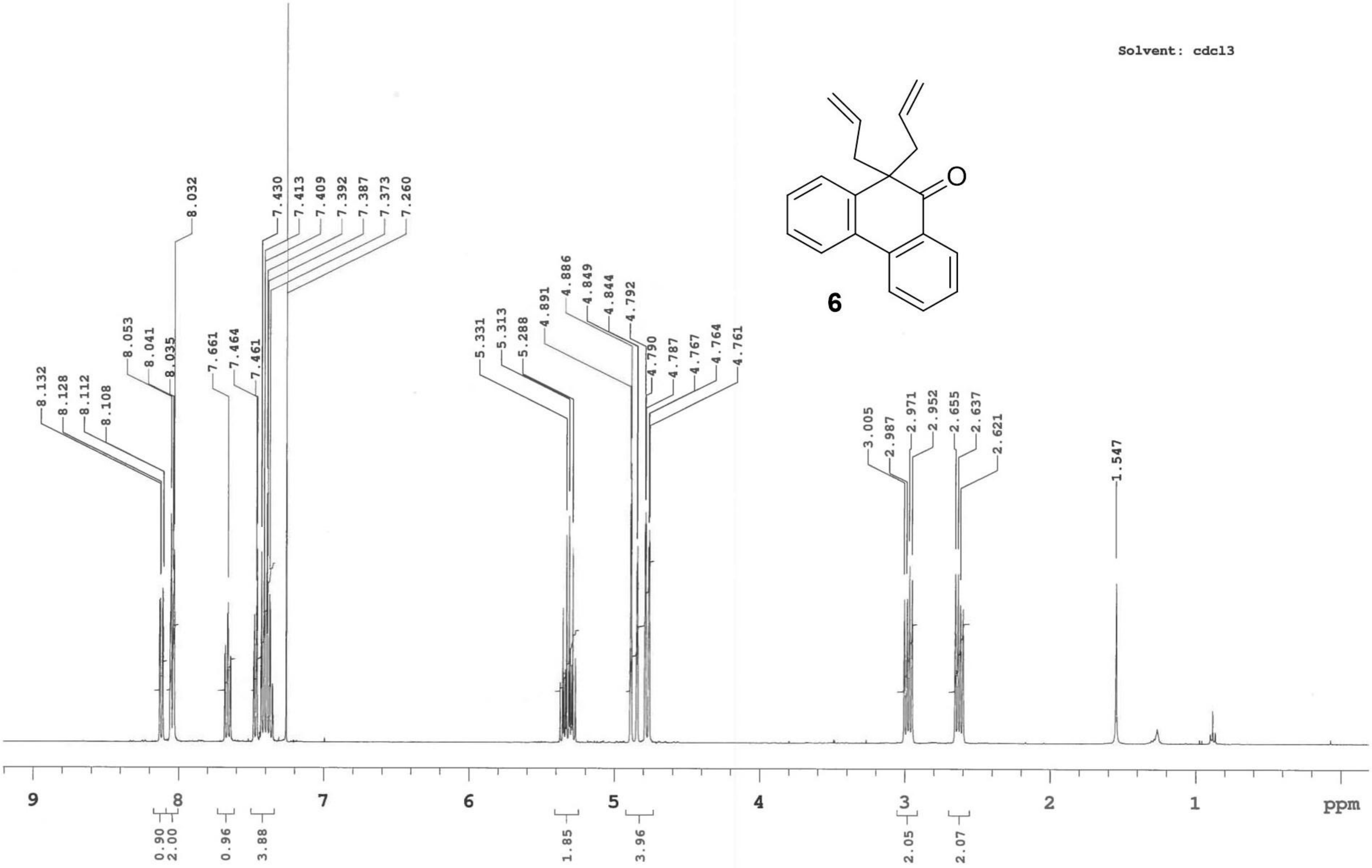
Solvent: cdcl3
6
8.132
8.128
8.112
8.108
8.053
8.041
8.035
8.032
7.661
7.464
7.461
7.430
7.413
7.409
7.392
7.387
7.373
7.260
5.331
5.313
5.288
4.891
4.886
4.849
4.844
4.792
4.790
4.787
4.767
4.764
4.761
3.005
2.987
2.971
2.952
2.655
2.637
2.621
1.547
9
8
7
6
5
4
3
2
1
ppm
0.90
2.00
0.96
3.88
1.85
3.96
2.05
2.07

Solvent: cdcl3

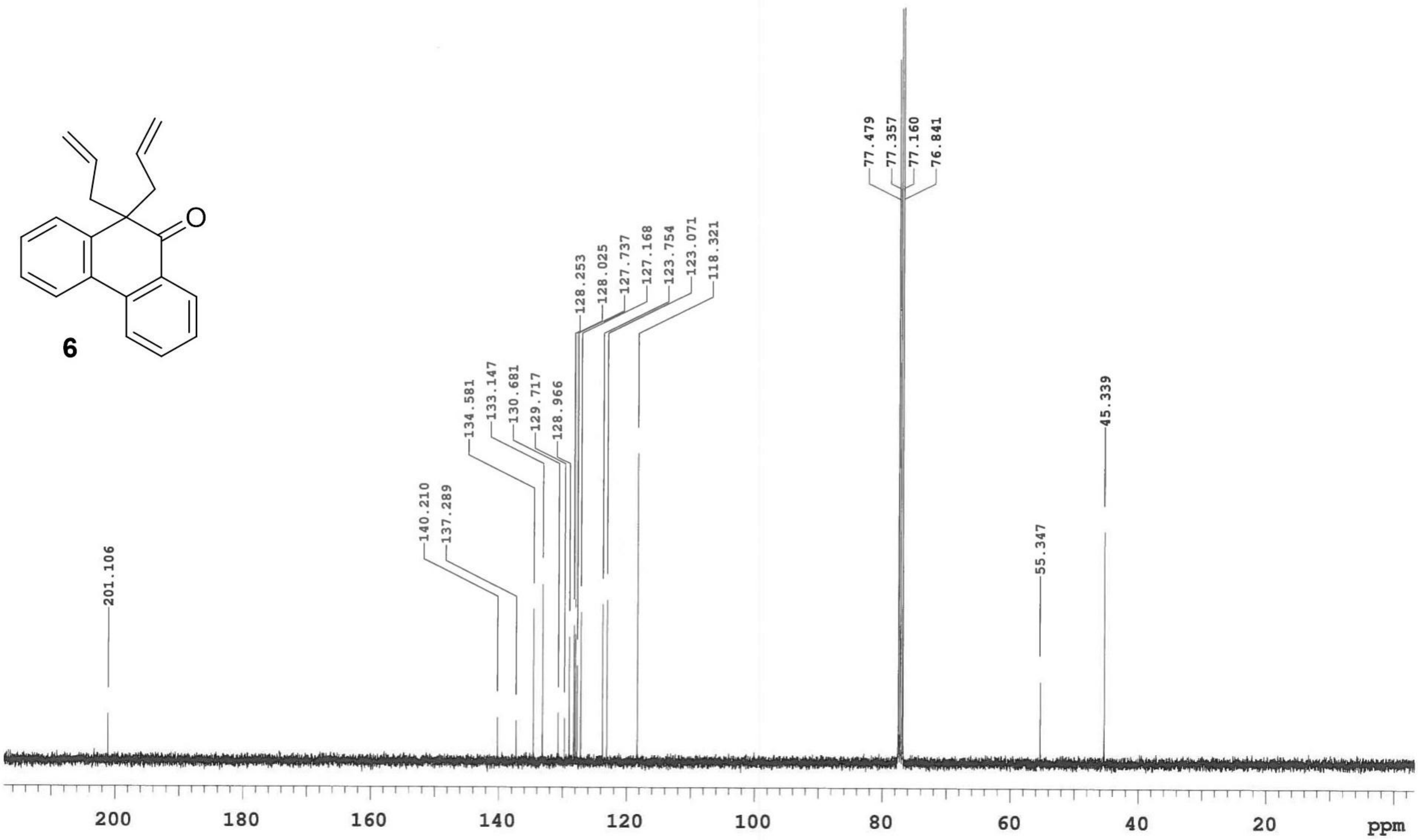

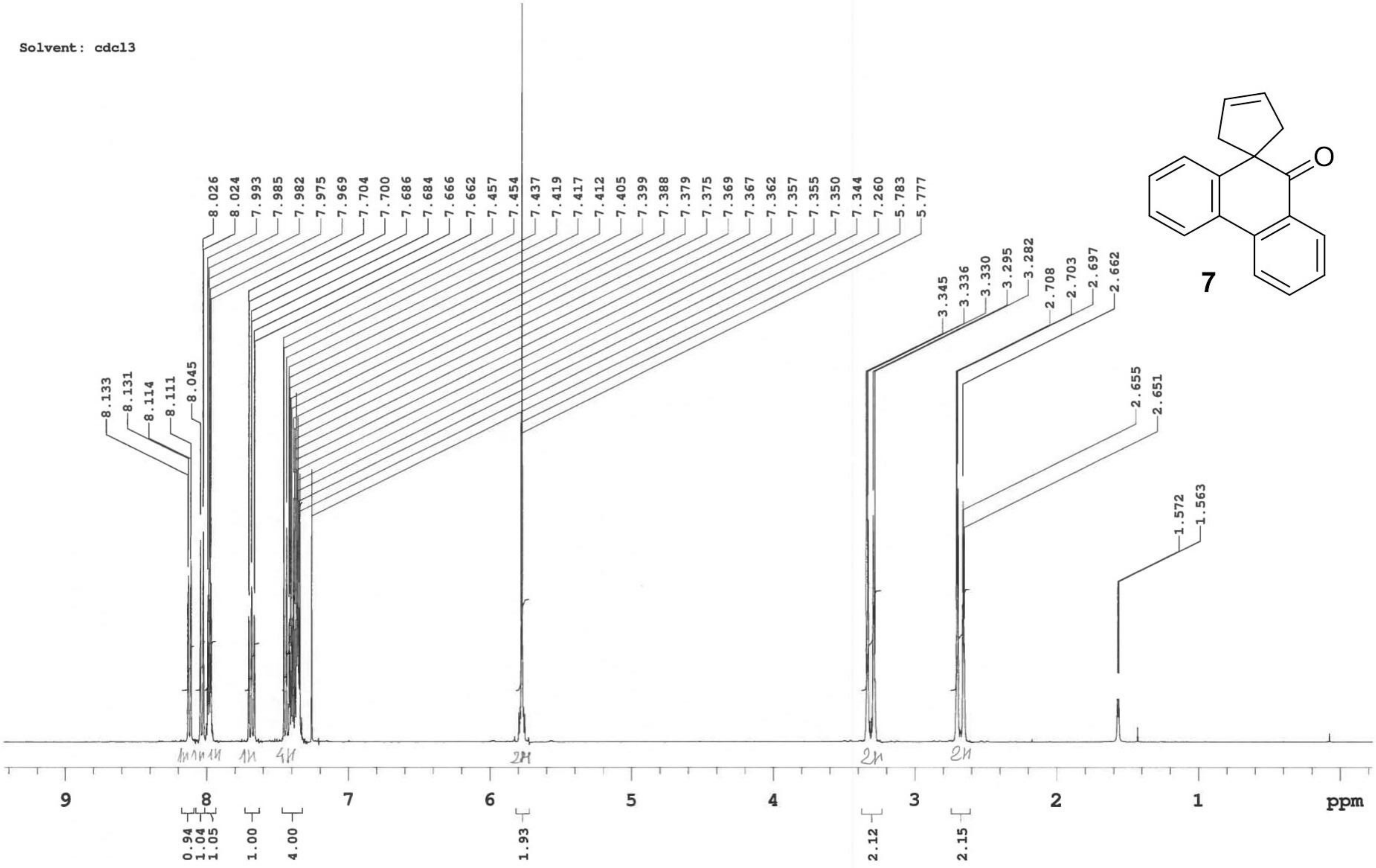
Solvent: cdcl3
7
8.133
8.131
8.114
8.111
8.045
8.026
8.024
7.993
7.985
7.982
7.975
7.969
7.704
7.700
7.686
7.684
7.666
7.662
7.457
7.454
7.437
7.419
7.417
7.412
7.405
7.399
7.388
7.379
7.375
7.369
7.367
7.362
7.357
7.355
7.350
7.344
7.260
5.783
5.777
3.345
3.336
3.330
3.295
3.282
2.708
2.703
2.697
2.662
2.655
2.651
1.572
1.563
9
8
7
6
5
4
3
2
1
ppm
0.94
1.04
1.05
1.00
4.00
1.93
2.12
2.15

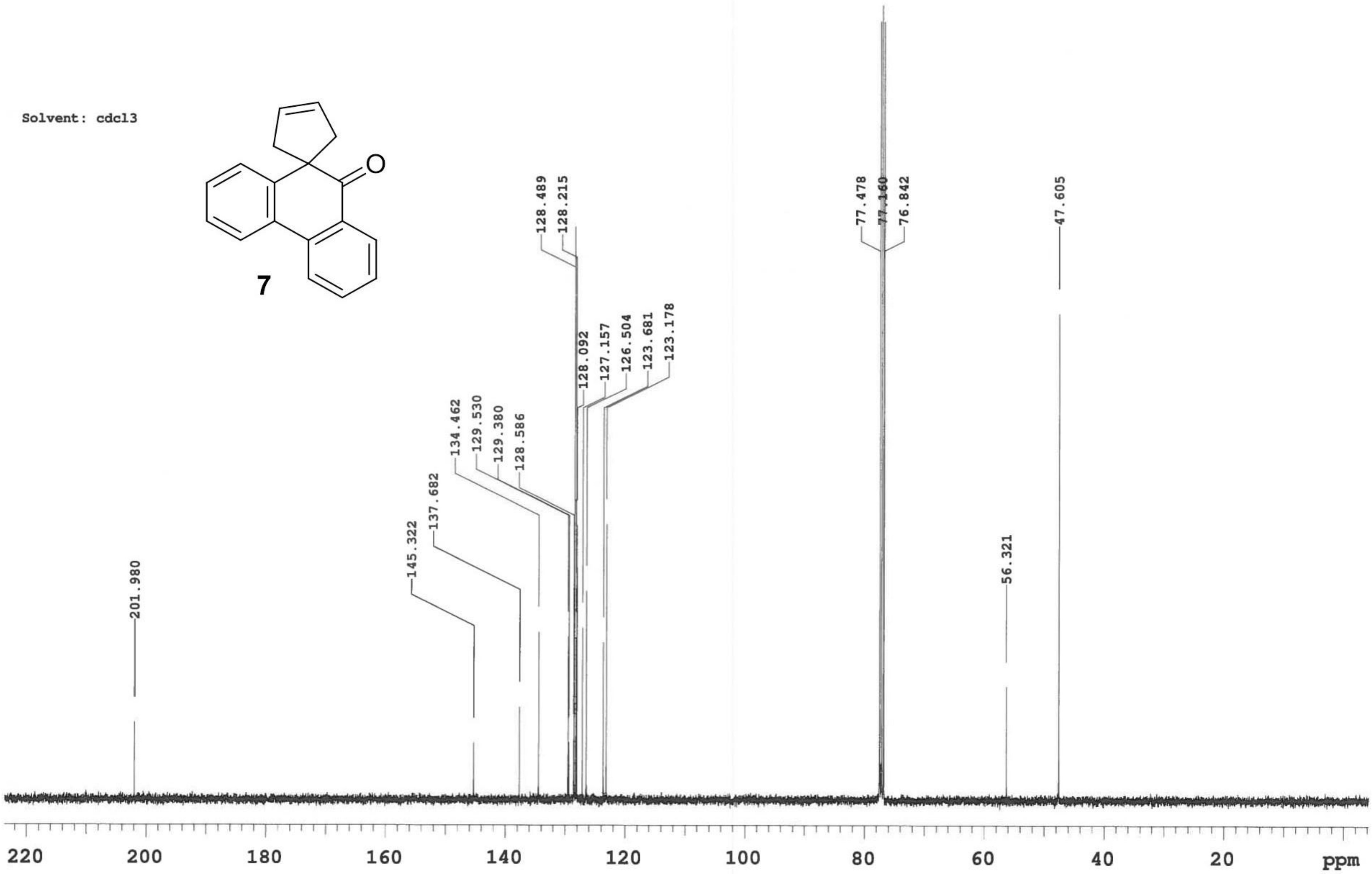
Solvent: cdcl3
7
201.980
145.322
137.682
134.462
129.530
129.380
128.586
128.489
128.215
128.092
127.157
126.504
123.681
123.178
77.478
77.160
76.842
56.321
47.605
220
200
180
160
140
120
100
80
60
40
20
ppm

Solvent: dmso
Temp. 90.0 C / 363.1 K

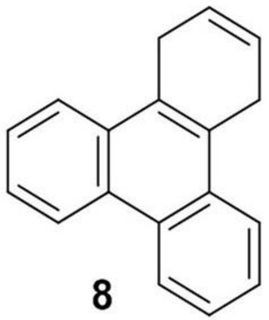

8

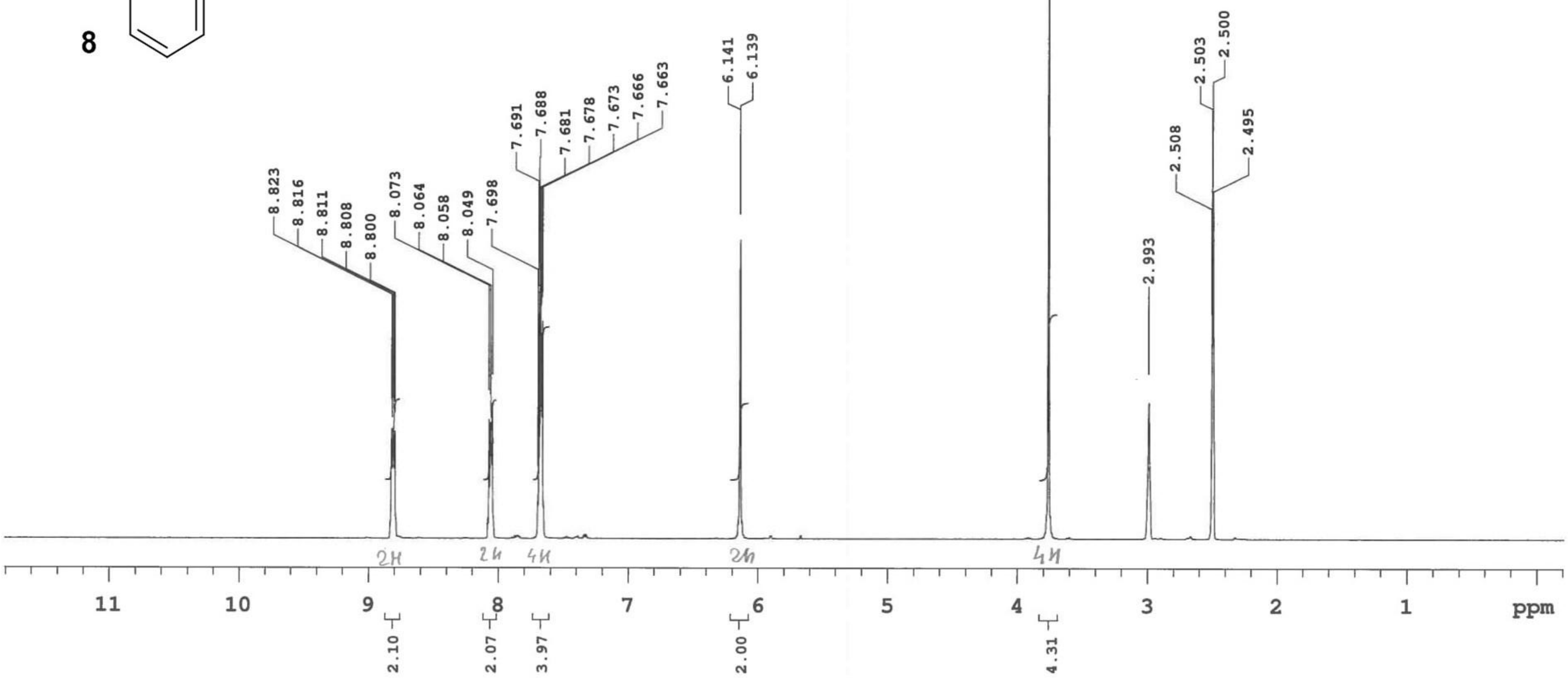

Solvent: dmso
Temp. 90.0 C / 363.1 K

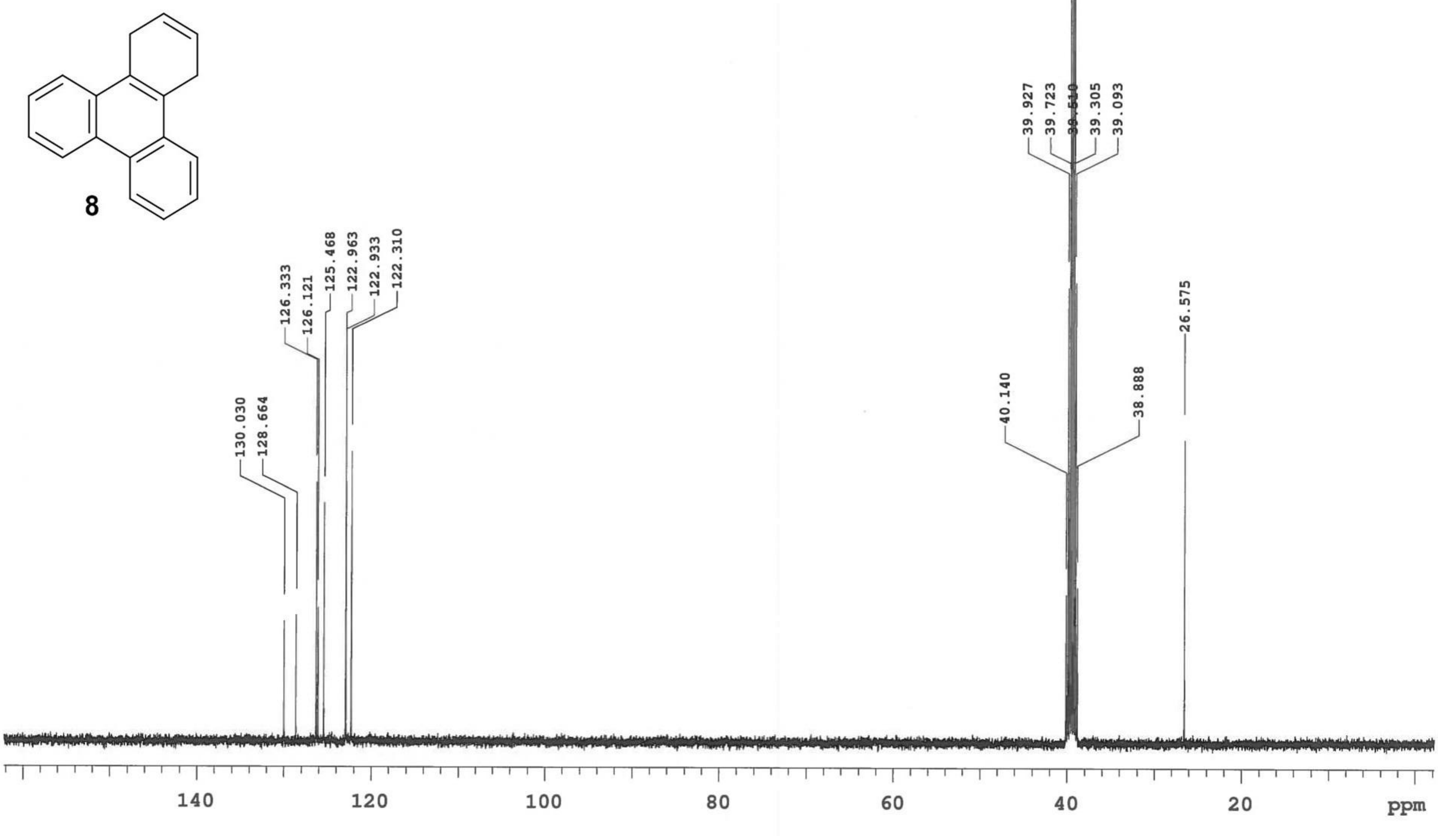

Solvent: cdcl3

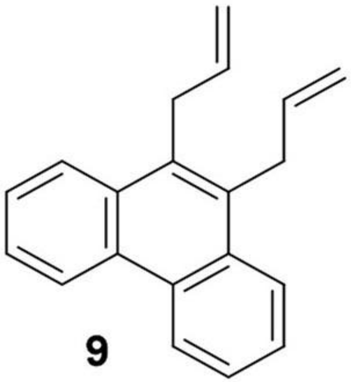

9

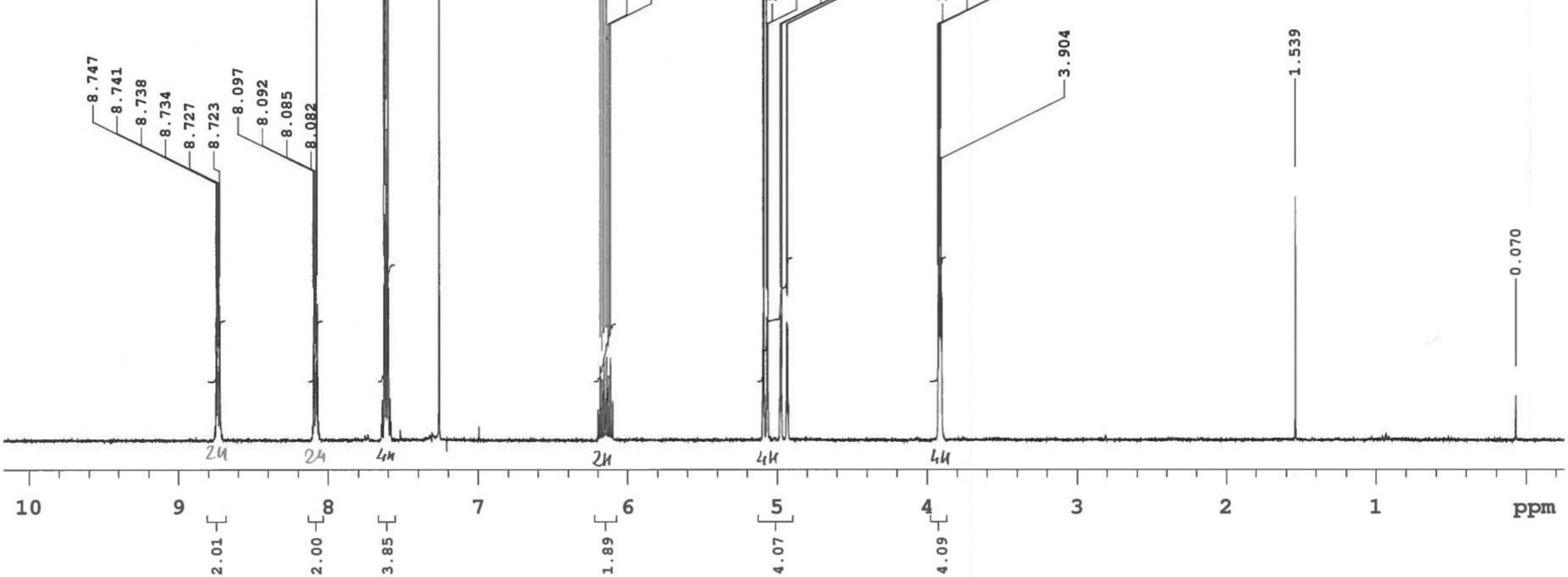

Solvent: cdcl3

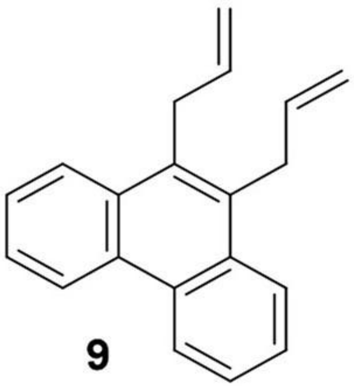

9

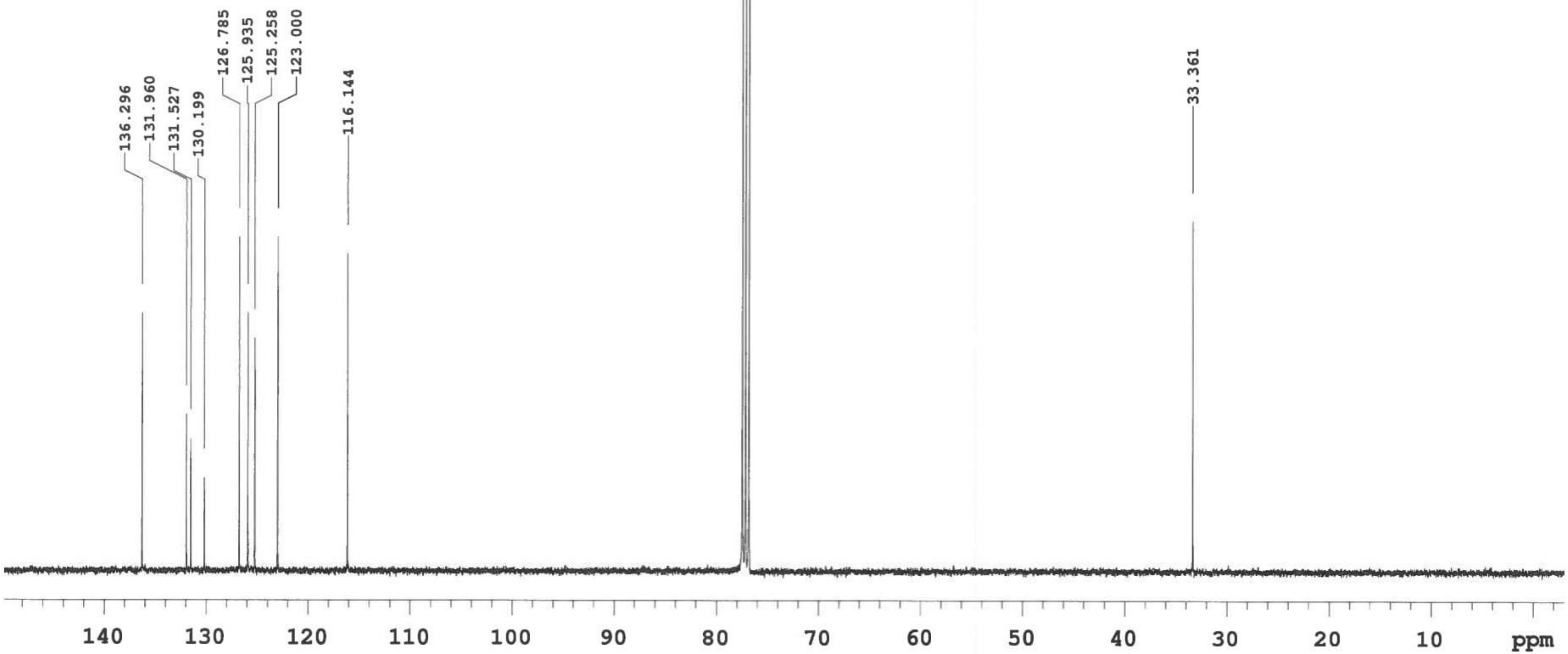

Supplement: Supplementary file 1 [file molecules-23-02043-s001.pdf]
